# *Helicobacter pylori*, Inflammation, and Long‐Term Outcome in Patients With Acute Myocardial Infarction: A Prospective Cohort Study

**DOI:** 10.1111/hel.70116

**Published:** 2026-03-15

**Authors:** Martin O. Sundqvist, Jonatan Wärme, Marcus Hjort, Per Tornvall, Tomas Jernberg, Bertil Lindahl, Alexandru Schiopu, Tomasz Baron, Stefan H. Jacobson, Thomas Kahan, David Erlinge, Jonas Spaak, Robin Hofmann

**Affiliations:** ^1^ Department of Clinical Science and Education, Södersjukhuset Karolinska Institutet Stockholm Sweden; ^2^ Department of Cardiology Södersjukhuset Stockholm Sweden; ^3^ Department of Medical Sciences and Uppsala Clinical Research Center Uppsala University Uppsala Sweden; ^4^ Division of Cardiovascular Medicine, Department of Clinical Sciences, Danderyd Hospital Karolinska Institutet Stockholm Sweden; ^5^ Uppsala Clinical Research Center and Department of Medical Sciences Uppsala University Uppsala Sweden; ^6^ Department of Translational Medicine Lund University Malmö Sweden; ^7^ Department of Internal Medicine Skåne University Hospital Lund Sweden; ^8^ Institute of Cellular Biology and Pathology “N. Simionescu” Bucharest Romania; ^9^ Division of Nephrology, Department of Clinical Sciences Karolinska Institutet Danderyd Hospital Stockholm Sweden; ^10^ Department of Cardiology, Clinical Sciences Lund University Lund Sweden

**Keywords:** biomarkers, coronary heart disease, *Helicobacter pylori*, inflammation, myocardial infarction

## Abstract

**Background:**

*Helicobacter pylori* (Hp) and its virulence factor Cytotoxin‐associated gene A (CagA) have been linked to myocardial infarction (MI), but the mechanisms are unknown. This study aims to test if Hp infection and CagA are associated with pre‐specified inflammatory and vascular biomarkers in patients with MI and to explore whether a broader biomarker panel can predict infection. Furthermore, it aims to investigate the association of Hp infection and biomarkers with major adverse cardiovascular events (MACE) and mortality.

**Materials and Methods:**

Hp, CagA serology, and 175 cardiovascular biomarkers were analyzed in 1061 patients with MI admitted between 2008 and 2014. Associations between Hp and seven pre‐selected biomarkers were evaluated. Exploratory analyses included all biomarkers using machine‐learning models to predict Hp‐status. Hp‐status and the top predictors were analyzed for associations with outcomes using Cox regression.

**Results:**

Median age was 65 years; 78% were male. Hp and CagA seroprevalence were 45% and 19%, respectively. Patients with Hp had elevated CRP (*β* = 0.26, 95% CI 0.01–0.51). Predictive performance of Hp‐status was moderate (AUC 0.63–0.68). Exploratory analysis identified higher levels of C‐C motif chemokine ligand 20 (CCL20) and immunoglobulin heavy constant gamma‐3 (IGHG3), and lower levels of TNF‐related apoptosis‐inducing ligand (TRAIL) in patients with Hp‐positivity. Elevated CCL20 and reduced TRAIL, but not Hp, were associated with MACE and all‐cause mortality.

**Conclusions:**

Hp may contribute to an inflammatory response in patients with MI, indicated by higher CRP and inflammatory/immune‐modulatory biomarkers emerging as its top predictors. Although Hp was not associated with adverse outcomes after MI, its predictive inflammatory biomarkers were associated with MACE and mortality.

**Trial Registration:**

The study was not registered as a clinical trial, as it was an observational study

## 1 Introduction

Several pathogens have been linked to the development of cardiovascular disease and myocardial infarction (MI) [1]. Specifically, *Helicobacter pylori* ( *H. pylori* ) may cause chronic infection of the gastric and duodenal mucosa and has been associated with atherosclerosis and MI [2]. The mechanisms underlying this relationship remain undetermined. Postulated mechanisms include immune activation with systemic inflammation, induction of dyslipidaemia, molecular mimicry with antibody cross‐reactivity, prothrombotic effects, endothelial dysfunction, and increased arterial stiffness [3, 4]. Additionally, different *H. pylori* strains and virulence factors may affect this relationship. The Cytotoxin‐associated gene A (CagA) is a major virulence factor that increases the risk of gastric malignancy and severe ulcers [5]. CagA‐positive *H. pylori* strains have a stronger association with MI [6], and CagA has been detected in exosomes in the systemic circulation of patients infected with *H. pylori* [7]. Experimental studies show that mice infected with CagA‐positive *H. pylori* secrete extracellular vesicles containing CagA, which can reach the distal vasculature [8]. CagA protein induces vascular smooth muscle cell calcification in vitro [9]. This suggests a potential direct mechanism linking *H. pylori* to MI and other vascular diseases, further supported by studies revealing *H. pylori* residues and CagA within atherosclerotic plaques [10, 11].

Previous studies have associated *H. pylori* with increased inflammation [12] and lower high‐density lipoprotein (HDL) cholesterol levels in healthy individuals [13], and eradication treatment may reduce C‐reactive protein (CRP) and improve lipid profile [14, 15, 16]. In patients with established cardiovascular disease, the association between *H. pylori* and vascular and inflammatory biomarkers is not well described, with previous work limited to small cohorts and few biomarkers [17]. Patients with unstable angina with positive CagA serology have been reported to have higher CRP levels [18], and patients with MI and *H. pylori* have higher neutrophilic inflammation compared with healthy controls [19]. However, several confounding factors exist between *H. pylori* and cardiovascular diseases [20], increasing the risk of bias.

Since *H. pylori* is a common, treatable infection that may amplify inflammatory and vascular dysfunction pathways linked to MI, defining its biomarker signature and association with subsequent major adverse cardiovascular events (MACE) could identify a modifiable contributor to residual risk after MI. This study aimed to test the hypothesis that *H. pylori* infection and CagA are associated with pre‐specified inflammatory and vascular biomarkers in patients with acute MI. Subsequently, we evaluated whether a comprehensive panel of 175 biomarkers could predict *H. pylori* infection status and identified those with the strongest predictive performance for *H. pylori* to explore potential pathological pathways. Lastly, we investigated whether *H. pylori* infection and related biomarkers were associated with MACE and all‐cause mortality following acute MI.

## 2 Materials and Methods

### 2.1 Study Population

The study population consisted of patients admitted for a suspected acute MI between March 2008 and September 2014 at three Swedish university hospitals in Stockholm, Uppsala, and Lund, which are included in the nationwide MI registry Swedish Web‐system for Enhancement and Development of Evidence‐based care in Heart disease Evaluated According to Recommended Therapies (SWEDEHEART) [21]. Patients were asked to donate a blood sample during the acute hospitalization to a biobank at the treating hospital (StockholmHeartBank, Uppsala SWEDEHEART biobank, and LUNDHEARTGENE). An acute MI was defined using International Classification of Diseases, 10th revision (ICD‐10) code (I21), based on the discharge diagnosis in the SWEDEHEART registry. Only blood samples from the first occurrence of MI during the study period were included for patients with repeated measurements. Patients were excluded in the case of non‐MI acute coronary syndrome or any other non‐type 1 MI diagnosis at discharge, incomplete biomarker data, or incomplete serology results for *H. pylori* and CagA. Written informed consent was obtained for blood sample collection and biobanking for later use in research. The study was conducted according to the principles of the Declaration of Helsinki and was approved by the Regional Ethical Review Board in Stockholm (2017/759–31), with an amendment by the Swedish Ethical Review Authority (2022–02810‐02).

### 2.2 Biomarker Analysis

Peripheral blood was collected by venous puncture at fasting in the morning in EDTA tubes (BD Vacutainer) or 3.2% citrate tubes on days 1–3 after admission. Samples were immediately brought to the laboratory and centrifuged at 4°C within 20 min and stored in plasma aliquots at −80°C. Sampling was carried out at a single time point for each patient. Biomarker concentrations were determined using Proximity Extension Assay (PEA) and Multiple Reaction Monitoring (MRM). The PEA measured 92 cardiovascular biomarkers with the Olink Proseek Multiplex CVD I96 × 96 kit (Olink Bioscience, Uppsala, Sweden) using paired antibodies and subsequent real‐time PCR to quantify proteins, as previously described [22, 23]. Results were analyzed as normalized protein expression units, using log_2_‐transformed values, which produce relative measurement values. Measurements with concentrations under the lower limit of detection (LOD) were replaced with the LOD value. This method has high reproducibility and repeatability with a mean intra‐assay coefficient of variation of 8% [22].

Next, an MRM mass spectrometry assay panel analyzed concentrations of 87 cardiovascular protein biomarkers [24, 25]. Protein targets from the MRM assay were quantified using concentration‐balanced stable isotope standards (SIS) and log 2‐transformed to enable comparison with PEA concentrations. The mean intra‐assay coefficient of variation was 4.7% [24]. Biomarkers for both assays were preselected based on their potential relevance to cardiovascular disease, and their biological function in cardiovascular disease has previously been categorized and described [26]. In brief, biomarkers included markers of inflammation, coagulation, endothelial function, lipid metabolism, kidney function, and atherogenesis, most of which have been indicated as possible links between *H. pylori* and cardiovascular diseases [3, 27]. Seven biomarkers were preselected that were of particular interest for a possible association between *H. pylori* and MI. These included biomarkers indicative of systemic inflammation, which has been suggested as a possible mechanism by which *H. pylori* may contribute to MI [3]. CRP, interleukin‐6 (IL‐6), and lipopolysaccharide‐binding protein (LBP) were selected for this purpose. In addition, urokinase‐type plasminogen activator receptor (uPAR) was included as a biomarker of inflammation, as this was associated with *H. pylori* seropositivity in one of our previous studies [28]. The remaining biomarkers were selected to reflect markers of vascular calcification, as *H. pylori* has been associated with higher coronary artery calcification scores in cohort studies [29], and coronary artery vascular smooth muscle cell calcification after CagA induction in vitro [9]. For this purpose, fibroblast growth factor 23 (FGF‐23), osteoprotegerin (OPG), and nuclear factor‐κB essential modulator (NEMO) were selected. All biomarkers were then considered for exploratory analysis. After excluding biomarkers with few samples with concentrations above the LOD (interleukin‐4) and isoforms of apolipoprotein E, as well as selecting one peptide per protein where multiple peptides were analyzed, 175 biomarkers (91 from the PEA assay and 84 from the MRM assay) were included in the analysis, as previously described [24, 25]. The estimated glomerular filtration rate was calculated using creatinine measurements during hospitalization and the CKD‐EPI formula.

### 2.3 *H. pylori* and CagA Analysis

Anti‐ *H. pylori* IgG antibodies were measured using a commercial ELISA kit (Abcam, Helsinki, Finland, no. ab108736) according to the manufacturer's instructions. The recommended cut‐off of > 20 enzyme immune units per milliliter was used to define positive samples. One test was performed per sample. Positive samples were further analyzed with a commercial anti‐CagA IgG ELISA kit (Ravo Diagnostika Freiburg, Germany, no. HP004) according to the manufacturer's instructions. Samples with optical density values > 0.3 were considered positive as recommended by the manufacturer. One test was performed per sample. *H. pylori* negative samples were not analyzed for CagA and were considered CagA negative. *H. pylori* and CagA serology results were categorized into a dichotomous variable of *H. pylori* , and further into a three‐level variable that also included CagA status of *H. pylori* ‐positive samples ( *H. pylori* ‐positive/CagA‐positive, *H. pylori* ‐positive/CagA‐negative, *H. pylori* ‐negative).

### 2.4 Follow‐Up and Outcomes

Outcome variables were obtained for the study population by linking the SWEDEHEART registry to the Swedish National Patient Register and the Cause of Death Register for ICD‐10 codes from subsequent hospitalisations after the index event, as well as information regarding the cause of death. Held by the National Board of Health and Welfare, both registries have virtually complete coverage nationally and are considered to have good validity for cardiovascular diseases [30, 31]. Data regarding all‐cause mortality and MACE were retrieved for all patients after initial hospitalization for MI. MACE was defined as the composite of non‐fatal MI (ICD‐10 code I21), ischemic stroke (ICD‐10 code I63), or cardiovascular death (ICD‐10 code I00–I99 as the primary diagnosis for cause of death), and patients were censored in the case of non‐cardiovascular death. Patients were followed from discharge until 16 May 2018 for all‐cause mortality, and until 31 December 2017 for MACE composites. In the Swedish National Patient Register, it is not possible to distinguish a readmission connected to the index MI from a new, recurrent event during the first 30 days after discharge after an MI event. Thus, only new MI events after 30 days could be assessed.

### 2.5 Statistical Methods

Numerical variables were reported as medians and interquartile ranges, and categorical variables were reported as frequencies and percentages. Clinical baseline variables were compared between *H. pylori* groups using a two‐tailed Student's *t*‐test for numerical variables and a Chi‐square test for categorical variables.

The seven prespecified biomarkers were analyzed using multiple linear regression with biomarker concentration as the dependent variable and *H. pylori* and CagA status as covariates. A crude model and an adjusted model were fitted, adjusting for age, sex, smoking, estimated glomerular filtration rate, body mass index, and recorded diagnoses of hypertension, diabetes mellitus and hyperlipidaemia, according to our perception of a possible causal relationship between *H. pylori* and biomarker concentrations (Figure S1). Results were analyzed for all patients who were *H. pylori‐*positive, and with the CagA group in the three‐level variable. Beta coefficients for *H. pylori* and CagA were presented with 95% confidence intervals. Prespecified subgroups were sex and infarct type based on diagnosis of non‐ST‐elevation MI or ST‐elevation MI. Biomarker analysis was performed after dividing the data into these subgroups, with results presented separately for each group. No adjustment for multiple testing was performed in this step. Two post hoc analyses were carried out. First, a sensitivity analysis evaluated models additionally adjusted for medications at admission that could affect biomarker concentrations by adding the use of angiotensin‐converting enzyme inhibitors or angiotensin receptor blockers on admission. Second, a subgroup analysis evaluated ejection fraction during hospitalization as a proxy for infarct size, categorized by left ventricular ejection fraction over and under 40%.

In the exploratory analysis, random forest and lasso machine learning regression models were trained for the classification of *H. pylori* status using all available biomarkers and clinical data, which was compared to a regular logistic regression model. The data was split into 80% training data and 20% testing data. Five‐fold cross‐validation was used due to the relatively small size of the dataset. Model training and evaluation were performed using the Tidymodels R package. For random forest, the number of trees was set to 500, while the number of predictors for each split (mtry) and the minimum number of data points per split (min n) were selected using tuning, which considers a range of different hyperparameter values and selects the optimal values based on performance. For lasso regression, the shrinkage parameter was also selected using tuning, and the shrinkage parameter was chosen based on the best area under the curve (AUC) value. Model performance was visualized and evaluated using receiver operating characteristic (ROC) curves and AUC values. The most important variables and biomarkers used for prediction were obtained from the final models of random forest and lasso using a variable importance score for random forest and by calculating odds ratios of non‐shrunken variables in lasso. Variables with the 10 highest variable importance scores or largest odds ratio deviations from one were presented for the random forest and lasso models, respectively. A false discovery rate‐adjusted *p*‐value was additionally applied to account for all 175 biomarkers being used in the initial exploratory analysis, with a significance level set to 0.05.

Outcomes were assessed in survival analysis using Cox regression for *H. pylori* status and biomarkers. Separate models were fit for dichotomised *H. pylori* status and for *H. pylori* status including CagA groups, and models were adjusted according to Figure S2. Biomarkers associated with *H. pylori* in pre‐specified and exploratory analyses were divided into tertiles to study outcomes based on tertile levels with adjustments according to Figure S3. The proportional hazards assumption was evaluated using Schoenfeld residuals for each covariate included in the models. Violations were controlled by using stratification for categorical variables and time transformation for numerical variables. Results were graphically presented as adjusted cumulative incidence plots using direct standardization.

Missing data for clinical variables and biomarkers are presented in Table S1. As there were few missing values overall, single imputation was used to complete each missing data point using the Mice package. All biomarkers and clinical data were considered for use in imputation using the quickpred function, which selects variables with a minimum Pearson correlation of 0.1 to the target variable. Imputation was then performed with logistic regression as models for categorical variables and predictive mean matching for numerical variables. The significance level was set at an alpha of 0.05. Statistical analyses were performed using R version 4.2.3 (R Foundation for Statistical Computing, Vienna, Austria).

## 3 Results

**FIGURE 1:**
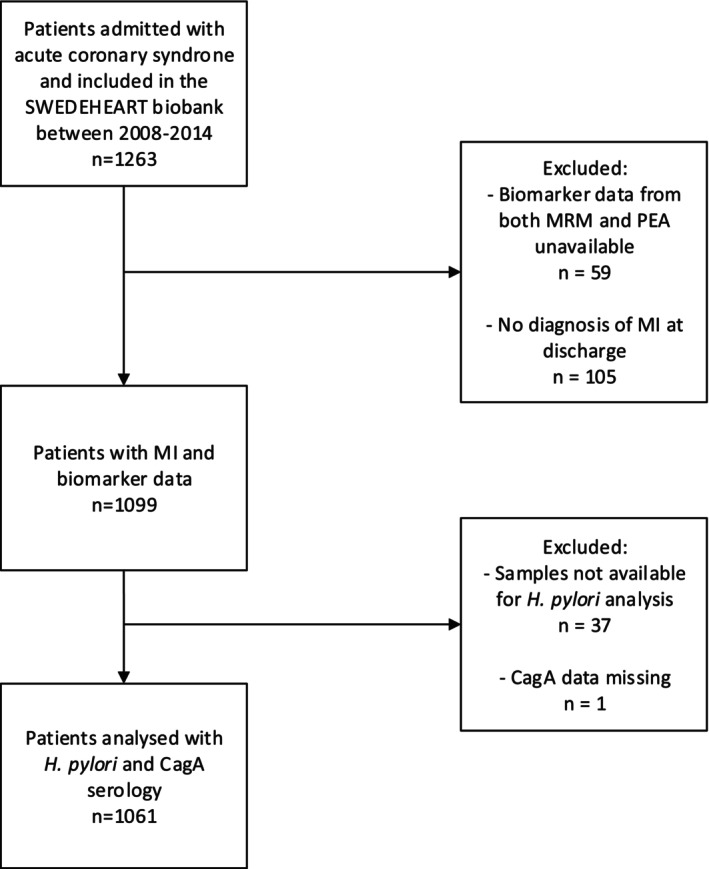
Flowchart for biomarker, *H. pylori* , and CagA analysis in patients with MI. MI, Myocardial infarction; CagA, Cytotoxin‐associated gene A; MRM, Multiple Reaction Monitoring; PEA, Proximity Extension Assay; SWEDEHEART, Swedish Web‐system for Enhancement and Development of Evidence‐based care in Heart disease Evaluated According to Recommended Therapies.

After exclusion, 1061 patient samples were analyzed for *H. pylori* and CagA serology (Figure 1). Median age of the patients was 65 years, and 827 (78%) were male. In total, 475 (45%) of patients were seropositive for *H. pylori*, and 201 (19%) were seropositive for CagA (Table 1). *H. pylori‐*positive patients were older, had lower eGFR, and more often had a diagnosis of hyperlipidemia, heart failure, and previous PCI. They also had a higher use of statins and angiotensin‐converting enzyme inhibitors or angiotensin receptor blockers on admission. During hospitalization, both groups had similar left ventricular ejection fraction and received treatment with PCI and CABG to the same extent.

**TABLE 1:** Baseline characteristics of patients by *H. pylori* infection status.

	*H. pylori* positive (*N* = 475)	*H. pylori* negative (*N* = 586)	*p*
Sex, male	363 (76.4%)	464 (79.2%)	0.28
Age Median (Q1, Q3)	67.0 (59, 73)	63.0 (56, 71)	< 0.01
Smoking	130 (27.4%)	166 (28.3%)	0.73
BMI kg/m^2^	26.9 (24.7, 29.4)	26.9 (24.6, 29.4)	0.78
eGFR mL/min/1.73 m^2^	82.5 (64.5, 94.1)	86.4 (70.0, 96.2)	< 0.01
Prior diagnoses			
Hypertension	231 (48.6%)	303 (51.7%)	0.32
Diabetes mellitus	361 (76.0%)	459 (78.3%)	0.37
Hyperlipidemia	161 (34.1%)	148 (25.4%)	< 0.01
MI	75 (15.8%)	87 (14.8%)	0.67
PCI	82 (17.3%)	70 (11.9%)	0.01
CABG	40 (8.4%)	36 (6.1%)	0.15
Heart failure	71 (14.9%)	61 (10.4%)	0.03
Stroke	39 (8.2%)	46 (7.8%)	0.83
Peripheral artery disease	26 (5.5%)	19 (3.2%)	0.07
Previous cancer	12 (2.5%)	12 (2.0%)	0.60
COPD	31 (6.5%)	33 (5.6%)	0.54
Dementia	1 (0.2%)	0 (0.0%)	0.27
Medications before admission			
Aspirin	159 (33.7%)	133 (22.8%)	< 0.01
Other antiplatelet	30 (6.4%)	33 (5.7%)	0.65
Anticoagulants	18 (3.8%)	21 (3.6%)	0.86
Beta‐blockers	152 (32.4%)	178 (30.7%)	0.56
ACE‐i or ARB	161 (34.3%)	160 (27.6%)	0.02
Statins	159 (33.7%)	140 (24.1%)	< 0.01
Hospitalization course			
LVEF			
< 30%	24 (5.6%)	21 (4.0%)	0.43
30%–39%	61 (14.3%)	83 (15.7%)	
40%–49%	90 (21.1%)	98 (18.6%)	
> 50%	251 (58.9%)	326 (61.7%)	
PCI	404 (85.1%)	498 (85.0%)	0.97
CABG	22 (4.6%)	28 (4.8%)	0.91
MI type			
STEMI	228 (48.0%)	298 (50.9%)	0.36
NSTEMI	247 (52.0)	288 (49.1%)	
Angiography findings			
Normal/atheroma	27 (5.8%)	29 (5.1%)	0.19
Single‐vessel disease	191 (41.3%)	269 (47.0%)	
Multi‐vessel disease	244 (52.8%)	274 (47.9%)	
Medications at discharge			
Aspirin	457 (96.6%)	563 (96.7%)	0.92
Other antiplatelet	429 (90.9%)	539 (92.6%)	0.31
Anticoagulants	45 (9.5%)	54 (9.3%)	0.90
Beta‐blockers	433 (91.5%)	526 (90.4%)	0.51
ACE‐i or ARB	405 (85.6%)	475 (81.6%)	0.08
Statins	454 (96.0%)	563 (96.7%)	0.51

*Note:* Categorical variables are presented as *n* (%), and numerical variables are presented as median (Q1, Q3). Student's *t*‐test was used to test differences between numerical variables, and the Chi‐square was used for categorical variables.

Abbreviations: ACE‐i, Angiotensin‐converting enzyme inhibitor; ARB, Angiotensin receptor blocker; BMI, Body mass index; CABG, Coronary artery bypass grafting; *H. pylori* , *Helicobacter pylori* ; LVEF, Left ventricular ejection fraction; MI, Myocardial infarction; NSTEMI, Non‐ST‐elevation MI; PCI, Percutaneous coronary intervention; STEMI, ST‐elevation MI.

### 3.1 Prespecified Biomarker Analysis

**FIGURE 2:**
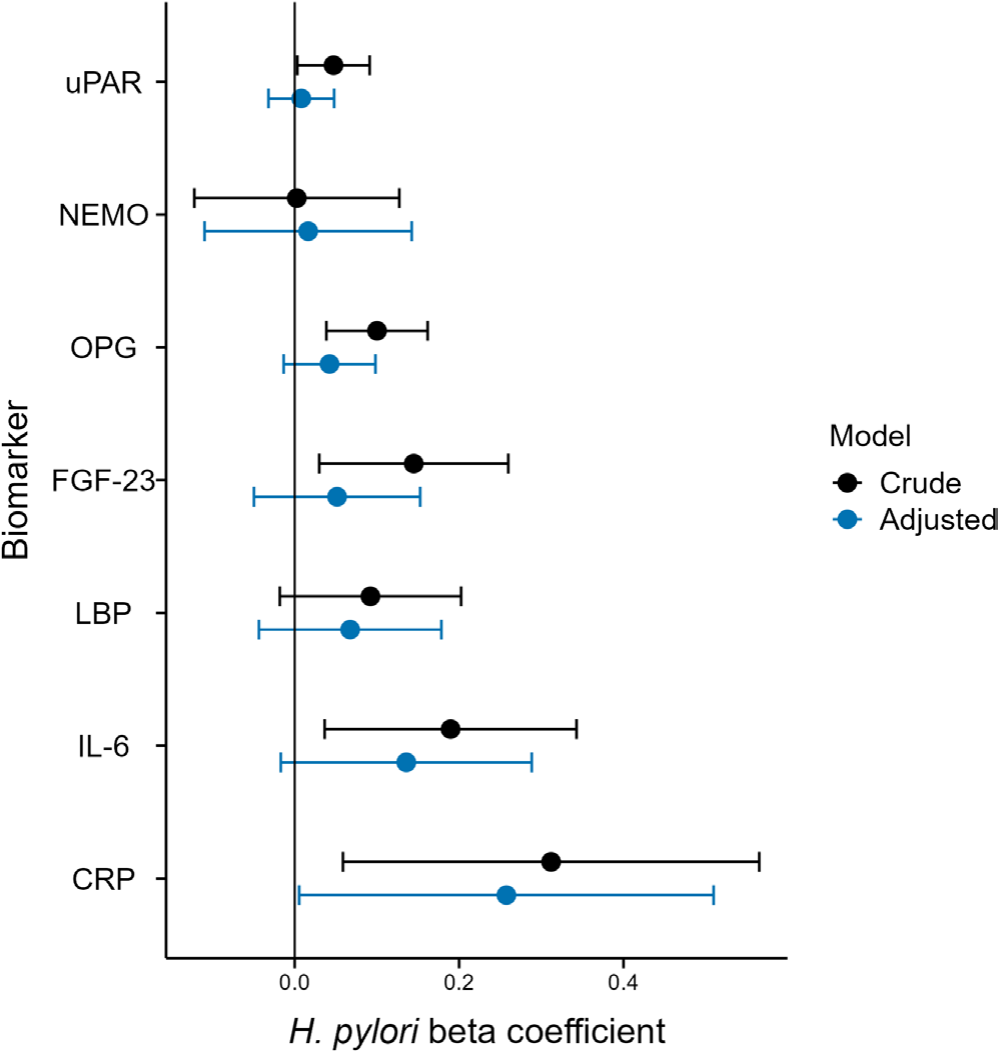
Biomarkers associated with *H. pylori* seropositivity in patients with myocardial infarction. Beta‐coefficients from the *H. pylori* positive group are displayed using linear regression for each prespecified biomarker with 95% confidence intervals. A crude model and an adjusted model are presented, which include sex, age, current smoking, hypertension, diabetes mellitus, eGFR, body mass index, and hyperlipidemia as covariates. uPAR, Urokinase‐type plasminogen activator receptor; NEMO, Nuclear factor‐κB essential modulator; OPG, Osteoprotegerin; FGF‐23, Fibroblast growth factor 23; LPB, Lipopolysaccharide‐binding protein; IL‐6, Interleukin‐6; CRP, C‐reactive protein.

The results from testing the hypothesis that *H. pylori* infection and its CagA‐positive subtype were associated with the prespecified biomarkers using linear regression are presented in Figure 2 for all patients positive for *H. pylori*, and in Figure S4 stratified by CagA status. In crude models, several biomarker concentrations were higher in the *H. pylori* ‐positive group compared to *H. pylori* ‐negative patients with MI, but the significance was lost for most biomarkers following multiple adjustments. CRP concentrations remained higher in patients who were *H. pylori* ‐positive (*β* = 0.26, 95% CI 0.01–0.51). When considering the CagA status for *H. pylori* , CRP concentration were higher in the CagA‐positive group specifically (*β* = 0.35, 95% CI 0.01–0.69). Median unadjusted, back‐transformed CRP concentrations were 5.6 mg/L in the *H. pylori* ‐negative group, 6.4 mg/L in the *H. pylori* ‐positive CagA‐negative group, and 7.9 mg/L in the CagA‐positive *H. pylori* ‐positive group.

Subgroup analyses by sex and infarction type are presented in Figures S5 and S6 for patients stratified by all *H. pylori‐*positive and CagA groups of *H. pylori* , respectively. The higher CRP levels of CagA‐positive *H. pylori* in the main analysis appear to be most pronounced in patients who were male and in patients with ST‐elevation MI. Furthermore, male patients with CagA‐positive *H. pylori* had significantly higher IL‐6 levels compared to their CagA‐negative counterparts. *H. pylori* or CagA positivity was not associated with higher IL‐6 levels in females. In a post hoc sensitivity analysis, the addition of adjustment for angiotensin‐converting enzyme inhibitors or angiotensin receptor blockers on admission, which were different at baseline between the groups, did not significantly alter the results (Figure S7). In the subgroup analysis of left ventricular ejection fraction above or below 40%, the main findings of higher CRP concentrations remained in the group with preserved ejection fraction, while there was no difference in the group under 40% (Figures S8 and S9). Additionally, LBP concentrations were higher in the *H. pylori* ‐positive group with left ventricular ejection fraction over 40%.

### 3.2 Exploratory Analyses and Prediction of *H. pylori* Status

Figure S10 shows the results of the final models used on testing data after training for the classification of *H. pylori* status using all available biomarkers and clinical data. The AUC value was highest for lasso regression, followed by regular logistic regression and random forest (lasso AUC 0.68, logistic regression AUC 0.65, random forest AUC 0.63). The top ten most important variables for the random forest and lasso models are shown in Table 2. C‐C motif chemokine ligand 20 (CCL20) and immunoglobulin heavy constant gamma 3 (IGHG3) were predictive biomarkers with high importance in both models. TNF‐related apoptosis‐inducing ligand (TRAIL) was the most impactful predictor in the lasso model with an OR of 0.70. To quantify the significance of the most important biomarkers, the five biomarkers showing the strongest associations from each model were further evaluated using linear regression, assessing beta‐coefficients and *p*‐values for the *H. pylori* covariate (Table S2). IGHG3, CCL20, and TRAIL had the lowest *p*‐values. To further test their significance and to account for multiple testing, a false discovery rate‐adjusted *p*‐value was applied, accounting for the testing of all 175 biomarkers. After adjustment, the same three biomarkers still retained a significant adjusted *p*‐value, while the remaining biomarker predictors were non‐significant.

**TABLE 2:** The ten most important variables for models to predict *H. pylori* status in patients with myocardial infarction.

Random forest variable	Importance	Lasso variable	Odds ratio
CCL20	100	TRAIL	0.70
IGHG3	60	IGHG3	1.28
Age	56	Adiponectin	0.80
IGHG2	51	No prior heart failure	0.81
IL‐6ra	27	CCL20	1.20
MBL2	22	Follistatin	1.18
MMP‐12	16	No aspirin on admission	0.85
Factor XII	15	Sex (female)	1.14
Aspirin on admission	14	No beta‐blockers on admission	1.14
CCL4	13	IL‐6ra	0.88

Abbreviations: CCL20, C‐C motif chemokine ligand 20; CCL4, C‐C motif chemokine ligand 4; IGHG2, Immunoglobulin heavy constant gamma 2; IGHG3, Immunoglobulin heavy constant gamma 3; IL6ra, Interleukin 6 receptor alpha; MBL2, Mannan‐binding lectin serine protease 2; MMP‐12, Matrix metallopeptidase 12; TRAIL, TNF‐related apoptosis‐inducing ligand.

### 3.3 *H. pylori* and Predictive Biomarkers on Outcomes After MI

*H. pylori* status and biomarkers that were associated with *H. pylori* positivity were used to study associations with clinical outcomes after MI. Patients had a mean follow‐up of 6.4 years for all‐cause mortality and 5.8 years for MACE. In total, 162 patients died, and 249 had a MACE. *H. pylori* and CagA serology were not associated with MACE, nor with all‐cause mortality (Table 3). Adjusted cumulative incidence plots are presented in Figure S11.

**TABLE 3:** Association between *H. pylori* and CagA serological status with outcomes after MI.

Group	MACE (*n* = 249)			All‐cause mortality (*n* = 162)		
	HR	95% CI	*p*	HR	95% CI	*p*
*H. pylori* negative (ref)						
*H. pylori* positive	1.13	[0.88–1.45]	0.352	0.99	[0.72–1.36]	0.937
*H. pylori* positive CagA negative	1.12	[0.83–1.52]	0.455	1.14	[0.79–1.66]	0.487
*H. pylori* positive CagA positive	1.14	[0.82–1.57]	0.440	0.83	[0.55–1.26]	0.388

*Note:* Cox regression models were used to evaluate HRs of MACE and all‐cause death after MI. Models were adjusted for sex, age, current smoking, hypertension, diabetes mellitus, eGFR, body mass index, hyperlipidemia, ST‐elevation MI or non‐ST‐elevation MI, year of hospitalization, and a previous diagnosis of chronic obstructive pulmonary disease, MI, heart failure, stroke, and gastrointestinal bleeding.

Abbreviations: CagA, Cytotoxin‐associated gene A; HR, Hazard ratio; MACE, Major adverse cardiovascular event.

Next, biomarkers that were associated with *H. pylori* in previous analyses were evaluated for outcomes after MI. In addition to CRP, biomarkers that remained significant after multiple testing adjustment in the exploratory analyses were evaluated (CCL20, TRAIL, and IGHG3). Results are presented in Table 4, and adjusted survival curves are presented in Figure S12 for MACE and Figure S13 for all‐cause mortality. For CCL20, the two higher concentration tertiles were both associated with MACE compared to the lowest one. The second‐level tertile was also associated with all‐cause mortality compared to the lowest one. Conversely, TRAIL concentrations were inversely associated with both MACE and all‐cause mortality, with more outcomes in the lowest concentration tertile. For CRP, the highest tertile was associated with all‐cause mortality, while IGHG3 was not associated with any adverse outcomes.

**TABLE 4:** Association between biomarker concentrations and outcomes after MI.

Biomarker tertiles	MACE (*n* = 249)			All‐cause mortality (*n* = 162)		
	HR	95% CI	*p*	HR	95% CI	*p*
CCL20 [2.24, 5.02] (ref)						
CCL20 [5.02, 5.83)	1.60	[1.13–2.25]	0.007	2.10	[1.33–3.32]	0.002
CCL20 [5.83, 12.06]	1.58	[1.11–2.26]	0.012	1.27	[0.78–2.06]	0.339
TRAIL [4.78, 6.78] (ref)						
TRAIL [6.78, 7.18)	0.80	[0.59–1.08]	0.138	0.73	[0.51–1.05]	0.093
TRAIL [7.18, 8.71]	0.73	[0.52–1.02]	0.066	0.40	[0.25–0.65]	0.001
IGHG3 [4.14, 8.05] (ref)						
IHGH3 [8.05, 8.81)	1.52	[1.1–2.09]	0.010	1.27	[0.84–1.91]	0.260
IGHG3 [8.81, 11.78]	1.34	[0.97–1.86]	0.080	1.42	[0.96–2.11]	0.083
CRP [−3.36, 1.64] (ref)						
CRP [1.64, 3.56)	1.19	[0.85–1.68]	0.308	0.88	[0.56–1.38]	0.580
CRP [3.56, 8.38]	1.40	[0.99–1.98]	0.056	1.57	[1.02–2.41]	0.039

*Note:* Biomarkers that were associated with *H. pylori* infection were categorized into tertiles and fitted into Cox regression models to evaluate HRs of MACE and all‐cause death on follow‐up. Models were adjusted for sex, age, current smoking, hypertension, diabetes mellitus, eGFR, body mass index, hyperlipidemia, year of hospitalization for MI, MI type, left ventricular ejection fraction, prior heart failure, angiotensin‐converting enzyme inhibitors or angiotensin receptor blockers on admission, prior stroke, prior MI and prior chronic obstructive pulmonary disease.

Abbreviations: CagA, Cytotoxin‐associated gene A; CCL20, C‐C motif chemokine ligand 20; HR, Hazard ratio; IGHG3, Immunoglobulin heavy constant gamma 3; MACE, Major adverse cardiovascular event; TRAIL, TNF‐related apoptosis‐inducing ligand.

A sensitivity analysis evaluated models accounting for non‐proportional hazards. The assumption was not violated by *H. pylori* or any biomarker, but for individual covariates. The results were only minimally affected after accounting for these violations (data not shown).

## 4 Discussion

In this study, using circulating biomarkers from patients with acute MI, prespecified analyses confirmed the hypothesis that *H. pylori* and CagA‐positive *H. pylori* are associated with inflammation, reflected by higher CRP levels. Six other inflammatory biomarkers showed increased levels in crude models but lacked statistical significance after adjustments. Exploratory analyses further supported this association by identifying three novel proteins, CCL20, IGHG3, and TRAIL, all involved in inflammation and immune response, as the most important predictors of *H. pylori* seropositivity. While *H. pylori* status was not correlated with adverse outcomes, higher CCL20 and lower TRAIL concentrations were linked to MACE and all‐cause mortality during follow‐up.

Previous studies have shown that *H. pylori* is associated with increased CRP levels in individuals without cardiovascular disease [12]. Whether similar findings can be seen in patients with MI has remained controversial. One study evaluated CRP, IL‐6, and four other inflammatory markers in relation to the serological status of *Chlamydia pneumoniae* , *H. pylori*, and cytomegalovirus in patients with acute coronary syndrome, but found no significant differences [17]. However, only 81 patients were included, of which 33 patients were seropositive for *H. pylori* . Another study reported no association between *H. pylori* serology and CRP levels in 53 patients with unstable angina [32], while one study found that CRP was higher among 204 patients with MI in the presence of both interleukin‐1 polymorphism and *H. pylori* positivity, but the comparison was unadjusted [33]. To the best of our knowledge, this study is the largest to date examining the association between *H. pylori* and inflammatory biomarkers in patients with acute MI. Additionally, a total of 175 cardiovascular biomarkers were studied, allowing exploratory analyses of pathways through which *H. pylori* may contribute to MI development.

In this study, higher concentrations of CRP, CCL20, IGHG3, and lower concentrations of TRAIL were associated with *H. pylori* infection in patients with MI. CRP is an established predictor of cardiovascular outcomes in both healthy individuals [34] and after MI [35]. The elevated CRP levels observed here may suggest increased systemic inflammation induced by *H. pylori* , a mechanism that has been commonly proposed [3]. The finding of higher CRP levels, specifically in the CagA‐positive group, could further support this, as CagA acts pro‐inflammatory due to its complex intracellular effects in the gastric epithelium [5]. The samples in this study were collected during hospitalization for MI, which inherently induces acute systemic inflammation. Consequently, the direct immunological effects of an underlying *H. pylori* infection may be more difficult to isolate using this study design. Notably, elevated CRP levels were not seen in the subgroup of patients with left ventricular ejection fraction below 40%, which may suggest that inflammation from a larger MI masked other chronic inflammatory effects. Nonetheless, the use of a large cohort of over 1000 samples and the within‐population analyses strengthen the validity of the observed associations between *H. pylori* seropositivity and elevated inflammatory biomarkers in patients with MI. Importantly, *H. pylori* serology is unlikely to be influenced by, or related to, the timing of sample collection during MI hospitalization, thereby minimizing potential confounding.

CCL20 is a chemokine involved in the immune response to *H. pylori* infection. *H. pylori* induces a localized gastric infection characterized by chronic neutrophilic inflammation that may persist even in asymptomatic individuals. A combined adaptive and innate immune response is induced, mediated by activation of nuclear factor‐κB, expression of chemokines and cytokines such as IL‐8 [36], and recruitment of T‐helper 1 and 17 cells. CCL20 expression is increased in *H. pylori‐*infected gastric mucosa, correlates with disease severity, and can be reversed by *H. pylori* eradication therapy [37]. Macrophages cultured with *H. pylori* also show increased CCL20 expression, potentially promoting Th17 cell differentiation via interaction with the CCL20 receptor [38, 39]. Beyond its role in gastric *H. pylori* infection, CCL20 has also been implicated in the development of atherosclerosis in experimental models [40], and higher systemic concentrations have been associated with increased risk of coronary artery disease in Mendelian randomization studies [41]. In this study, patients with MI and *H. pylori infection* had higher systemic CCL20 concentrations, which itself was associated with increased risk of MACE and all‐cause mortality. This finding suggests a possible link between chronic *H. pylori* infection, elevated CCL20 levels, and adverse cardiovascular outcomes. However, *H. pylori* status was not independently associated with MACE or all‐cause mortality. Consequently, the causal chain remains uncertain and needs to be evaluated in future studies.

TRAIL, an apoptosis marker, was significantly lower in patients with MI and *H. pylori* infection. This biomarker is also linked to the immune response to *H. pylori* infection, as *H. pylori* appears to increase cellular susceptibility to TRAIL‐mediated apoptosis in the gastric epithelium [39]. It is less clear if and how systemic levels are affected by *H. pylori* infection. However, TRAIL and its receptors are associated with atherosclerosis and cardiovascular disease. Lower TRAIL levels have been associated with increased all‐cause mortality [42], which is consistent with the results in this study. In contrast, a meta‐analysis showed conflicting results for this association [43]. Mechanistically, TRAIL inhibits vascular smooth muscle cell calcification in vitro, as reflected by increased vascular calcification in TRAIL knockout mice [44]. In line with this, patients with lower TRAIL concentrations had a higher risk of MACE in this study. If there is a link between *H. pylori* infection, the reduction in TRAIL, and adverse outcomes, this link remains ambiguous. The observed association in this study warrants further investigation.

Although *H. pylori* and CagA seropositivity were not directly associated with adverse outcomes in this study, inflammatory biomarkers linked to *H. pylori* infection were. This finding may suggest that not the infection itself, but rather the degree of inflammatory activity it can elicit, plays a more determining role. Alternatively, the association between *H. pylori* and biomarkers in this study may be too modest for *H. pylori* to be independently linked with adverse outcomes, even though the biomarkers themselves were. In addition, circulating biomarkers may reflect systemic inflammation but may not accurately reflect the pathological mechanisms of atherosclerosis. While a previous study found that CagA‐positive *H. pylori* is specifically associated with adverse cardiovascular events after acute coronary syndrome in a Japanese population [10], the effect appeared to depend on the presence of an interleukin‐1 polymorphism. Another study found that positive CagA serology was associated with MACE after STEMI in an Italian population [45]. Similar findings could not be replicated in this study, even though the CagA‐positive group had elevated CRP levels. Thus, differences in *H. pylori* strain characteristics, virulence factors, infection activity, and host susceptibility factors could also affect results and should be considered.

In a clinical context, the findings of this study raise the question of whether *H. pylori* eradication may resolve the immune and inflammatory activation caused by a chronic infection, and thereby reduce the risk of recurrent cardiovascular events after MI. While previous randomized trials have evaluated antibiotics for secondary prevention of MI, most trials did not specifically test for *H. pylori* or use adequate eradication therapy. The ROXIS trial showed a potential benefit in patients randomized to roxithromycin and a greater reduction in CRP, as compared with controls [46]. However, larger subsequent trials failed to show any benefits of antibiotics after MI, which was also concluded in a systematic review [47]. The STAMINA trial, however, found that *H. pylori* eradication reduced CRP levels in patients with unstable angina, while the effects were not significant in patients with MI [16]. The recently published HELP‐MI SWEDEHEART trial used a cluster randomized crossover design applied to 35 hospitals across Sweden to study the effect of routine *H. pylori* screening during the hospitalization period for acute MI on upper gastrointestinal bleeding [48]. The study intervention did not significantly reduce the risk of subsequent upper gastrointestinal bleeding after a median follow‐up of 1.9 years. Furthermore, cardiovascular outcomes were similar between the groups. However, the cardiovascular endpoints were secondary outcomes and were not included in the trial's power calculation. Long‐term effects on cardiovascular outcomes, following an extended follow‐up period, are planned to be reported in the future.

## 5 Strengths and Limitations

The main strength of this study was the large cohort size, including more than 1000 patients with MI, in addition to the extensive biomarker assays on 175 cardiovascular biomarkers and a structured long‐term follow‐up using high‐quality registry data. Furthermore, the prespecified analysis allowed hypothesis testing, limiting the risk of multiple testing errors, followed by an exploratory analysis. All analyses were adjusted for important confounders that may limit bias, even though the risk of residual confounding remains.

The main limitation of this study was the use of serology to determine *H. pylori* and CagA status, which cannot confirm whether the infection was active at the time of blood sampling. The 45% prevalence of *H. pylori* was also higher than expected in a Swedish population. We have previously reported that the prevalence of an active *H. pylori* infection in patients with MI is closer to 20% using the urea breath test, but that study might have underestimated the prevalence due to medications that can reduce test accuracy, such as proton pump inhibitors [49]. On the other hand, the serological approach in this study may have overestimated the prevalence by including previously eradicated individuals. Unfortunately, data on prior eradication treatment were not available.

Another limitation was that the study only included patients with MI and did not allow results to be compared with a control group or a group with other cardiovascular diseases. The differences in biomarker concentrations are observational, and despite adjustments, there is a risk of residual confounding, particularly due to both *H. pylori* and MI being associated with low socioeconomic status [20]. Furthermore, the PEA assay provides concentrations in relative values, which limits translation to clinical absolute cut‐off values. Results from exploratory analyses also require external validation. Finally, the exploratory analyses used machine learning models for variable selection, which necessitates external validation.

## 6 Conclusion

*H. pylori* and CagA serology were associated with increased inflammation in patients with acute MI, as indicated by higher CRP concentrations. Furthermore, the three most important predictors of *H. pylori* seropositivity—CCL20, IGHG3, and TRAIL—were all inflammatory or immune‐modulatory biomarkers. Although *H. pylori* status itself was not associated with adverse outcomes after MI, both CCL20 and TRAIL were associated with MACE and all‐cause mortality. These findings suggest that *H. pylori* may contribute to an inflammatory response in patients with MI, but it represents only one component within a complex inflammatory milieu. Future studies are needed to evaluate whether *H. pylori* eradication can improve cardiovascular outcomes.

## Author Contributions

Conceptualization: M.O.S., J.W., J.S., R.H. Data curation: M.O.S., J.W., R.H. Formal analysis: M.O.S., J.W. Funding acquisition: R.H., J.S., T.K. Investigation: M.O.S., J.W., P.T., T.J., B.L., A.S., T.B., S.H.J., T.K., D.E., J.S., R.H. Methodology: M.O.S., J.W., M.H., P.T., T.J., B.L., A.S., T.B., T.K., J.S., D.E., R.H. Resources: R.H., J.S., P.T., T.J., B.L., A.S., T.B., S.H.J., T.K., D.E., J.S. Supervision: R.H., J.S. Visualization: M.O.S. Writing – original draft: M.O.S. Writing – review and editing: M.O.S., J.W., M.H., T.J., B.L., A.S., T.B., S.H.J., T.K., D.E., J.S., R.H.

## Funding

The study was funded by the Swedish Research Council (2019‐00414), The Swedish Heart Lung Foundation (2021‐0275, 2024‐0419), and Region Stockholm (2020‐0314, 2022‐0674, 2010‐0289, 2012‐0517). R.H. was supported by the Swedish Heart Lung Foundation (2021‐0273) and the Region Stockholm (RS2021‐0933).

## Conflicts of Interest

R.H. reports lecture and advisory board fees to institution from MSD/Pfizer and AstraZeneca. J.S. reports lecture and advisory board fees from Bayer, AstraZeneca, Boehringer Ingelheim, Novo Nordisk, and Sanofi. S.H.H. reports payment for expert testimony from Boehringer Ingelheim, AstraZeneca, and Novo Nordisk.

## Supporting information


**Table S1:** Missing data.
**Table S2:** Linear regression evaluation of the top 5 biomarkers in Random Forest and Lasso models.
**Figure S1:** Directed acyclic graph of a possible causal relationship between *H. pylori* and cardiovascular biomarkers.
**Figure S2:** Directed acyclic graph of a possible causal relationship between *H. pylori* and adverse outcomes after MI.
**Figure S3:** Directed acyclic graph of a possible causal relationship between biomarkers and adverse outcomes after.
**Figure S4:** Biomarkers associated with *H. pylori* positivity in patients with myocardial infarction stratified by CagA status.
**Figure S5:** Subgroup analysis of preselected biomarkers by sex and myocardial infarction (MI) type in all *H. pylori* positive patients.
**Figure S6:** Subgroup analysis of preselected biomarkers by sex and myocardial infarction (MI) type in *H. pylori* positive patients stratified by CagA status.
**Figure S7:** Post hoc sensitivity analysis of prespecified biomarkers in patients with MI in *H. pylori* groups and cytotoxin‐associated gene A (CagA) groups, additionally adjusted for angiotensin‐converting enzyme inhibitors or angiotensin receptor blockers on admission.
**Figure S8:** Post hoc subgroup analysis of preselected biomarkers by left ventricular ejection fraction (LVEF) groups in *H. pylori* positive vs. negative patients with MI.1111.
**Figure S9:** Post hoc subgroup analysis of preselected biomarkers by left ventricular ejection fraction (LVEF) groups in patients with MI with *H. pylori* cytotoxin‐associated gene A (CagA) groups compared to *H. pylori* negative.
**Figure S10:** Prediction of *H. pylori* status in patients with myocardial infarction using biomarkers and clinical data.
**Figure S11:** Adjusted cumulative incidence plot of the association between *H. pylori* and CagA serology with Major adverse cardiovascular events (MACE) and all‐cause mortality after MI.
**Figure S12:** Adjusted cumulative incidence plot of the association between biomarkers that were different in *H. pylori* positive patients and major adverse cardiovascular events (MACE).
**Figure S13:** Adjusted cumulative incidence plot of the association between biomarkers that were different in *H. pylori* positive patients and all‐cause mortality.

## Data Availability

The data underlying this article cannot be shared publicly due to laws regarding the privacy of research participants and are therefore not made publicly available.
