# Supplementary material for: Helicobacter pylori, Inflammation, and Long‐Term Outcome in Patients With Acute Myocardial Infarction: A Prospective Cohort Study
Source: Helicobacter. 2026 Mar 15;31(2):e70116. doi: 10.1111/hel.70116 (PMC12989639; doi:10.1111/hel.70116)
Supplement: Supplementary file 1 — Table S1: Missing data. Table S2: Linear regression evaluation of the top 5 biomarkers in Random Forest and Lasso models. Figure S1: Directed acyclic graph of a possible causal relationship between H. pylori and cardiovascular biomarkers. Figure S2: Directed acyclic graph of a possible causal relationship between H. pylori and adverse outcomes after MI. Figure S3: Directed acyclic graph of a possible causal relationship between biomarkers and adverse outcomes after. Figure S4: Biomarkers associated with H. pylori positivity in patients with myocardial infarction stratified by CagA status. Figure S5: Subgroup analysis of preselected biomarkers by sex and myocardial infarction (MI) type in all H. pylori positive patients. Figure S6: Subgroup analysis of preselected biomarkers by sex and myocardial infarction (MI) type in H. pylori positive patients stratified by CagA status. Figure S7: Post hoc sensitivity analysis of prespecified biomarkers in patients with MI in H. pylori groups and cytotoxin‐associated gene A (CagA) groups, additionally adjusted for angiotensin‐converting enzyme inhibitors or angiotensin receptor blockers on admission. Figure S8: Post hoc subgroup analysis of preselected biomarkers by left ventricular ejection fraction (LVEF) groups in H. pylori positive vs. negative patients with MI.1111. Figure S9: Post hoc subgroup analysis of preselected biomarkers by left ventricular ejection fraction (LVEF) groups in patients with MI with H. pylori cytotoxin‐associated gene A (CagA) groups compared to H. pylori negative. Figure S10: Prediction of H. pylori status in patients with myocardial infarction using biomarkers and clinical data. Figure S11: Adjusted cumulative incidence plot of the association between H. pylori and CagA serology with Major adverse cardiovascular events (MACE) and all‐cause mortality after MI. Figure S12: Adjusted cumulative incidence plot of the association between biomarkers that were different in H. pylori positive patients and majo [file HEL-31-e70116-s001.docx]

***Helicobacter pylori* and Cardiovascular Biomarkers in Acute Myocardial Infarction - Supplementary material**

**Contents**

[Supplemental Table 1. Missing data of clinical variables and biomarkers. 2](#_Toc223597347)

[Supplemental Table 2. Linear regression evaluation of the top 5 biomarkers with the highest variable importance in random forest and the largest odds ratio deviation from one in Lasso used to predict *H. pylori* status in myocardial infarction patients. 3](#_Toc223597348)

[Supplemental Figure 1. Directed acyclic graph of a possible causal relationship between *H. pylori* and cardiovascular biomarkers. 4](#_Toc223597349)

[Supplemental Figure 2. Directed acyclic graph of a possible causal relationship between *H. pylori* and adverse outcomes after MI. MI: Myocardial infarction, BMI: Body mass index, COPD: chronic obstructive pulmonary disease. MACE: Major adverse cardiovascular outcome. eGFR: estimated glomerular filtration rate. 5](#_Toc223597350)

[Supplemental Figure 3. Directed acyclic graph of a possible causal relationship between biomarkers and adverse outcomes after MI. MI: Myocardial infarction, BMI: Body mass index, COPD: chronic obstructive pulmonary disease. MACE: Major adverse cardiovascular outcome. eGFR: estimated glomerular filtration rate, LVEF: Left ventricular ejection fraction, RAAS: Renin-Angiotensin-Aldosterone System 6](#_Toc223597351)

[Supplemental Figure 4. Biomarkers associated with *H. pylori* positivity in patients with myocardial infarction stratified by CagA status. 7](#_Toc223597352)

[Supplemental Figure 5. Subgroup analysis of preselected biomarkers by sex and myocardial infarction (MI) type in all *H. pylori* positive patients. 8](#_Toc223597353)

[Supplemental Figure 6. Subgroup analysis of preselected biomarkers by sex and myocardial infarction (MI) type in *H. pylori* positive patients stratified by CagA status. 9](#_Toc223597354)

[Supplemental Figure 7. Post-hoc sensitivity analysis of prespecified biomarkers in patients with MI in *H. pylori* groups and Cytotoxin-associated gene A (CagA) groups, additionally adjusted for angiotensin-converting enzyme inhibitors or angiotensin receptor blockers on admission. 10](#_Toc223597355)

[Supplemental Figure 8. Post-hoc subgroup analysis of preselected biomarkers by left ventricular ejection fraction (LVEF) groups in *H. pylori* positive vs negative patients with MI. 11](#_Toc223597356)

[Supplemental Figure 9. Post-hoc subgroup analysis of preselected biomarkers by left ventricular ejection fraction (LVEF) groups in patients with MI with *H. pylori* Cytotoxin-associated gene A (CagA) groups compared to *H. pylori* negative. 11](#_Toc223597357)

[Supplemental Figure 10. Prediction of *H. pylori* status in patients with myocardial infarction using biomarkers and clinical data. 12](#_Toc223597358)

[Supplemental Figure 11. Adjusted cumulative incidence plot of the association between *H. pylori* and CagA serology with Major adverse cardiovascular events (MACE) and all-cause mortality after MI. 13](#_Toc223597359)

[Supplemental Figure 12. Adjusted cumulative incidence plot of the association between biomarkers that were different in *H. pylori* positive patients and major adverse cardiovascular events (MACE). 14](#_Toc223597360)

[Supplemental Figure 13. Adjusted cumulative incidence plot of the association between biomarkers that were different in *H. pylori* positive patients and all-cause mortality. 15](#_Toc223597361)

| **Numbers missing** |  | **Percentage missing** |  |
| --- | --- | --- | --- |
| **Clinical variables** |  |  | |
| Hyperlipidemia | 6 | 0.6 | |
| BMI | 46 | 4.3 | |
| eGFR | 1 | 0.1 | |
| Prior heart failure | 2 | 0.2 | |
| Aspirin on admission | 6 | 0.6 | |
| Other antiplatelets on admission | 9 | 0.8 | |
| Anticoagulation on admission | 6 | 0.6 | |
| Beta-blockers on admission | 13 | 1.2 | |
| ACE-i or ARB on admission | 12 | 1.1 | |
| Statins on admission | 7 | 0.7 | |
| LVEF | 107 | 10.1 | |
| **Biomarkers** |  |  | |
| IGHG4 | 4 | 0.4 | |
| IGHM | 1 | 0.1 | |
| IGHA2 | 1 | 0.1 | |
| A1AG1 | 1 | 1 | |
| NEMO | 50 | 4.7 | |

Supplemental Table 1. Missing data of clinical variables and biomarkers. Only variables with missing data are displayed. BMI: Body mass index, eGFR: estimated glomerular filtration rate, ACE-i: angiotensin-converting enzyme inhibitor, ARB: Angiotensin receptor blocker, LVEF: Left ventricular ejection fraction, IGHG4: immunoglobulin heavy constant gamma 4, IGHM: immunoglobulin heavy constant mu, IGHA2: immunoglobulin heavy constant alpha 2, A1AG1: alpha-1-acid glycoprotein 1, NEMO: nuclear factor-κB essential modulator.

| Biomarker | Beta coefficient | P-value | Adjusted p-value |
| --- | --- | --- | --- |
| IGHG3 | 0.24028 | 0.00002 | 0.00264 |
| CCL20 | 0.26929 | 0.00003 | 0.00279 |
| TRAIL | -0.10366 | 0.00040 | 0.02352 |
| Adiponectin | -0.14960 | 0.00117 | 0.05109 |
| IGHG2 | 0.14689 | 0.00207 | 0.06984 |
| Follistatin | 0.09525 | 0.00759 | 0.18963 |
| IL6ra | -0.04901 | 0.08225 | 0.68542 |
| MBL2 | 0.04009 | 0.49362 | 0.98363 |

Supplemental Table 2. Linear regression evaluation of the top 5 biomarkers with the highest variable importance in random forest and the largest odds ratio deviation from one in Lasso used to predict *H. pylori* status in myocardial infarction patients. Biomarkers were evaluated using linear regression, and beta coefficients for *H. pylori* positivity are displayed in models adjusted for sex, age, smoking, hypertension, diabetes mellitus, eGFR, BMI, and hyperlipidemia. P-values are displayed unadjusted, as well as adjusted for multiple testing of all 175 available biomarkers using false-discovery rate. MBL2: Mannan-binding lectin serine protease 2, IGHG3: immunoglobulin heavy constant gamma 3, IGHG2: immunoglobulin heavy constant gamma 2, CCL20: C-C motif chemokine ligand 20, TRAIL: TNF-related apoptosis-inducing ligand, IL6ra: interleukin 6 receptor alpha.


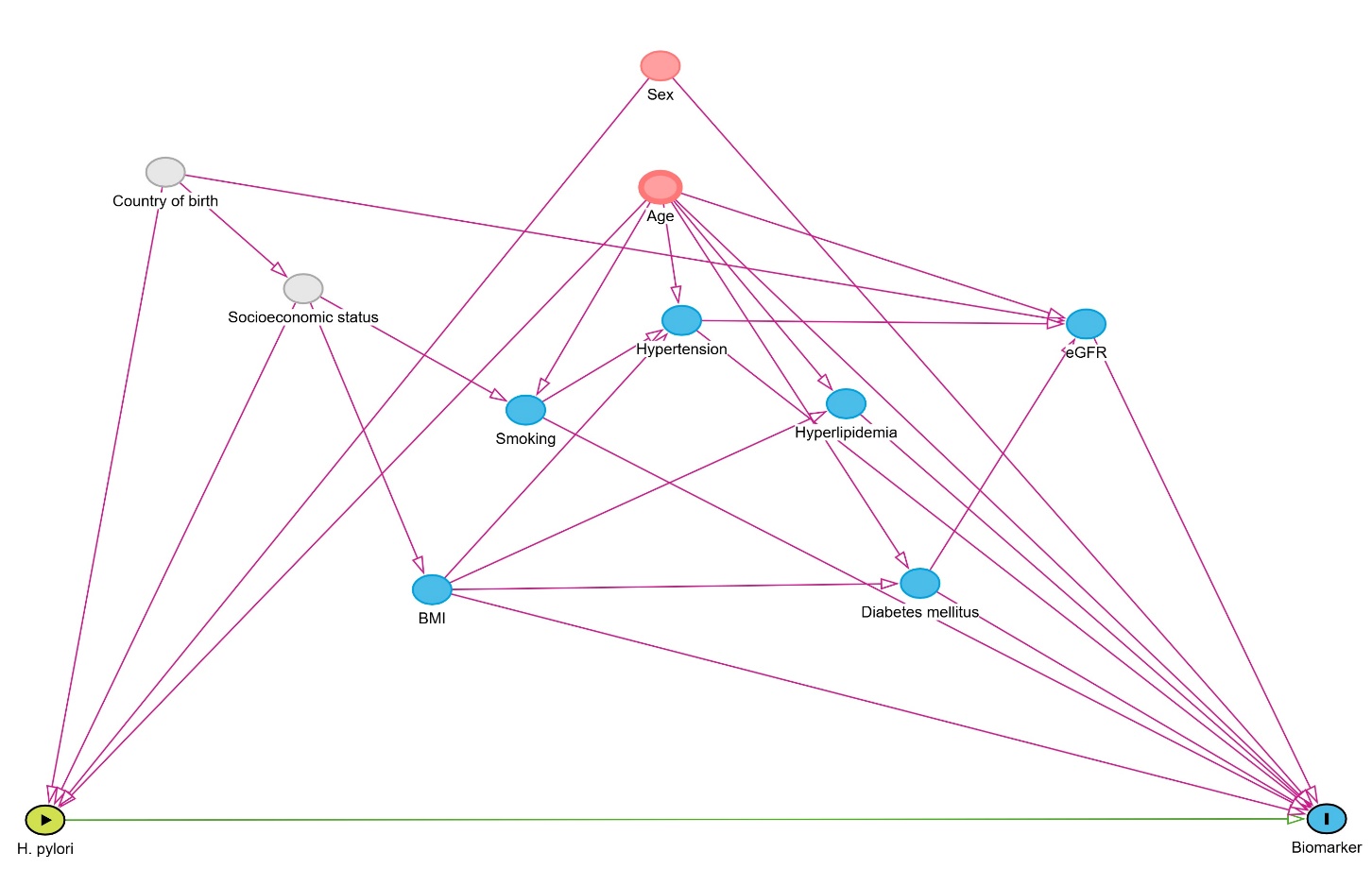


Supplemental Figure 1. Directed acyclic graph of a possible causal relationship between *H. pylori* and cardiovascular biomarkers. BMI: Body mass index, eGFR: estimated glomerular filtration rate.


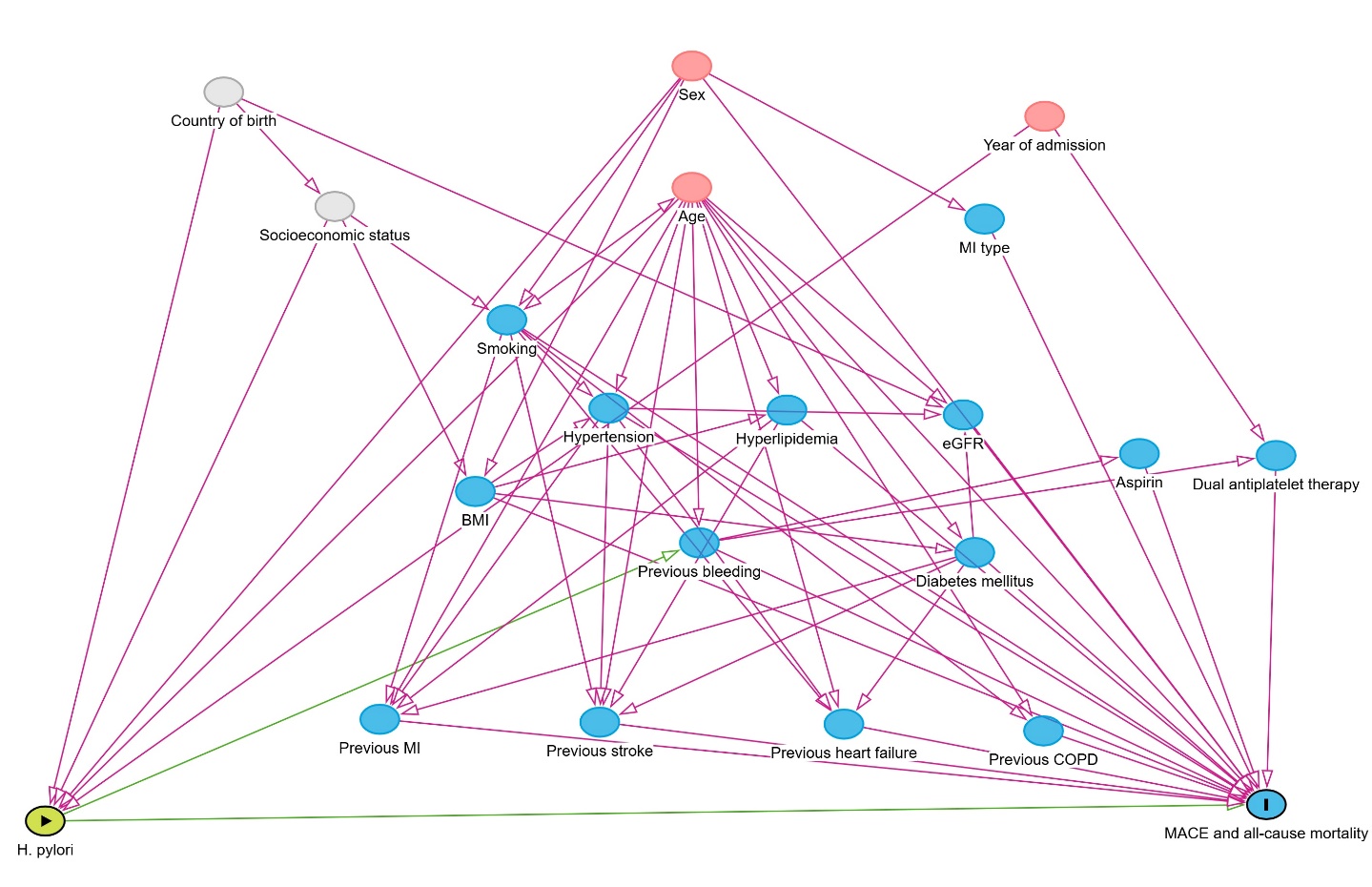


Supplemental Figure 2. Directed acyclic graph of a possible causal relationship between *H. pylori* and adverse outcomes after MI. MI: Myocardial infarction, BMI: Body mass index, COPD: chronic obstructive pulmonary disease. MACE: Major adverse cardiovascular outcome. eGFR: estimated glomerular filtration rate.


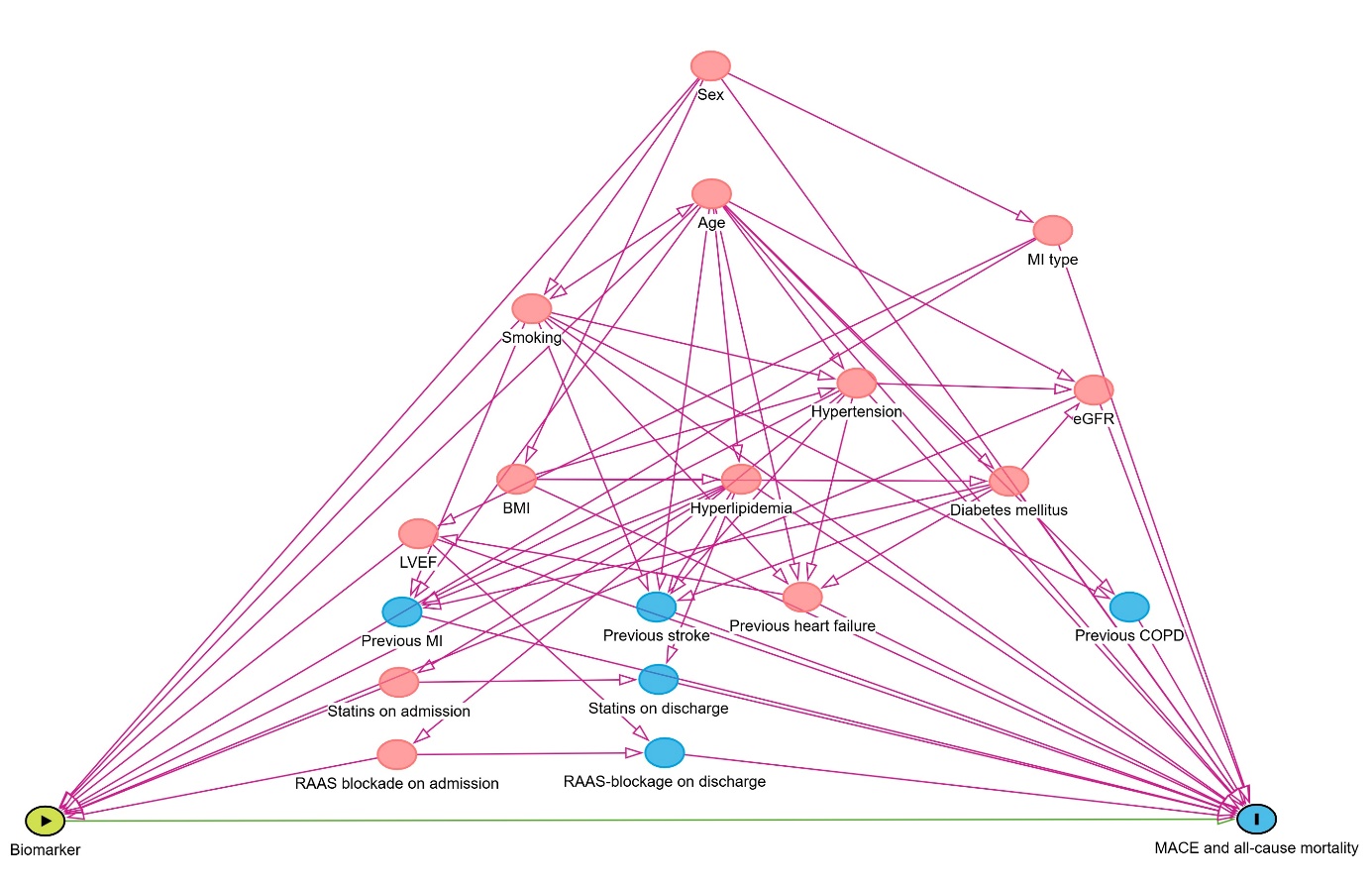


Supplemental Figure 3. Directed acyclic graph of a possible causal relationship between biomarkers and adverse outcomes after MI. MI: Myocardial infarction, BMI: Body mass index, COPD: chronic obstructive pulmonary disease. MACE: Major adverse cardiovascular outcome. eGFR: estimated glomerular filtration rate, LVEF: Left ventricular ejection fraction, RAAS: Renin-Angiotensin-Aldosterone System


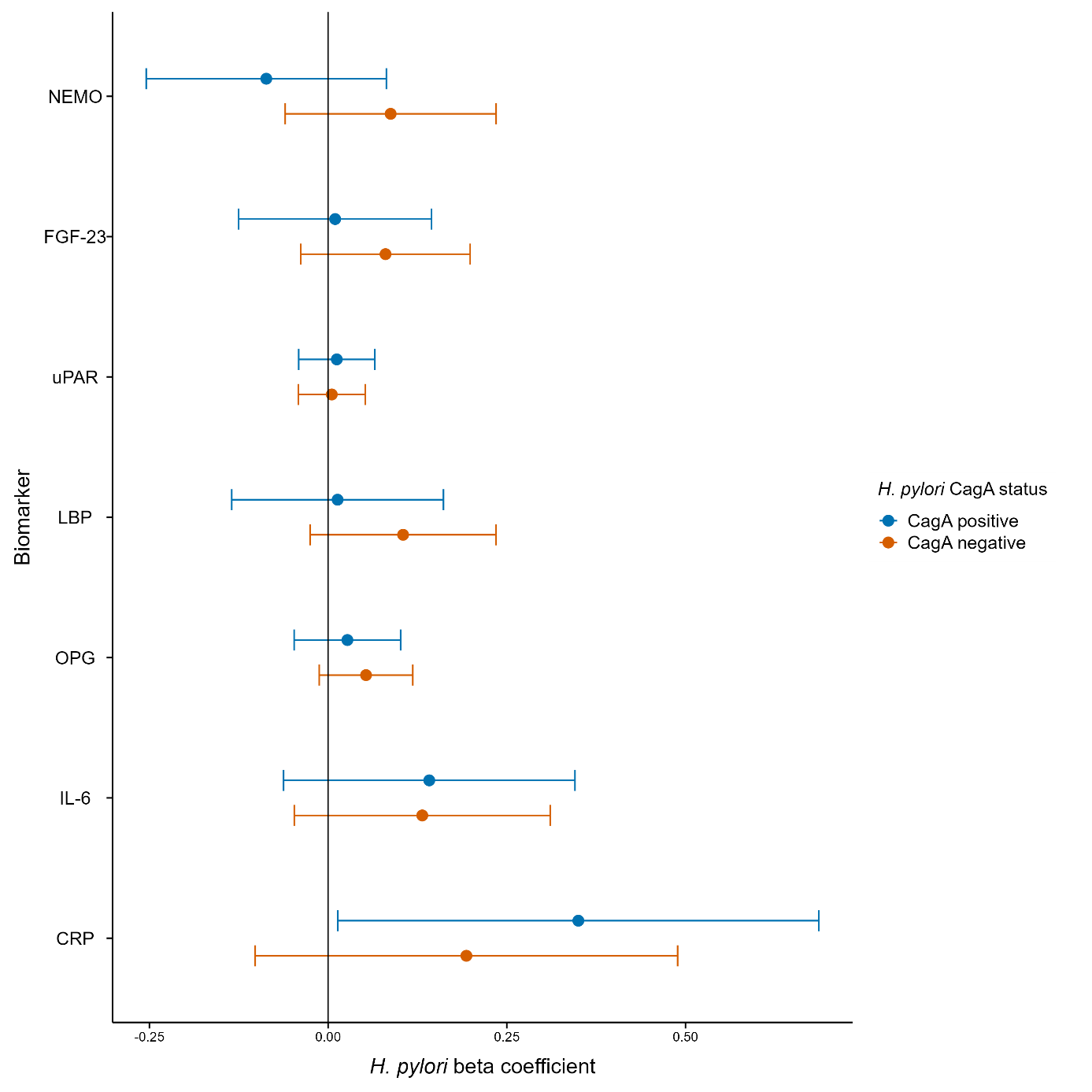


Supplemental Figure 4. Biomarkers associated with *H. pylori* positivity in patients with myocardial infarction stratified by CagA status. Beta-coefficients from the CagA status in the *H. pylori* positive group are displayed using linear regression for each pre-specified biomarker with 95% confidence intervals. Models were adjusted for sex, age, smoking, hypertension, diabetes mellitus, eGFR, body mass index, and hyperlipidemia. uPAR: urokinase-type plasminogen activator receptor, NEMO: nuclear factor-κB essential modulator. OPG: osteoprotegerin, FGF-23: Fibroblast growth factor 23, LPB: lipopolysaccharide binding protein, IL-6: interleukin-6, CRP: C-reactive protein, CagA: Cytotoxin-associated gene A.


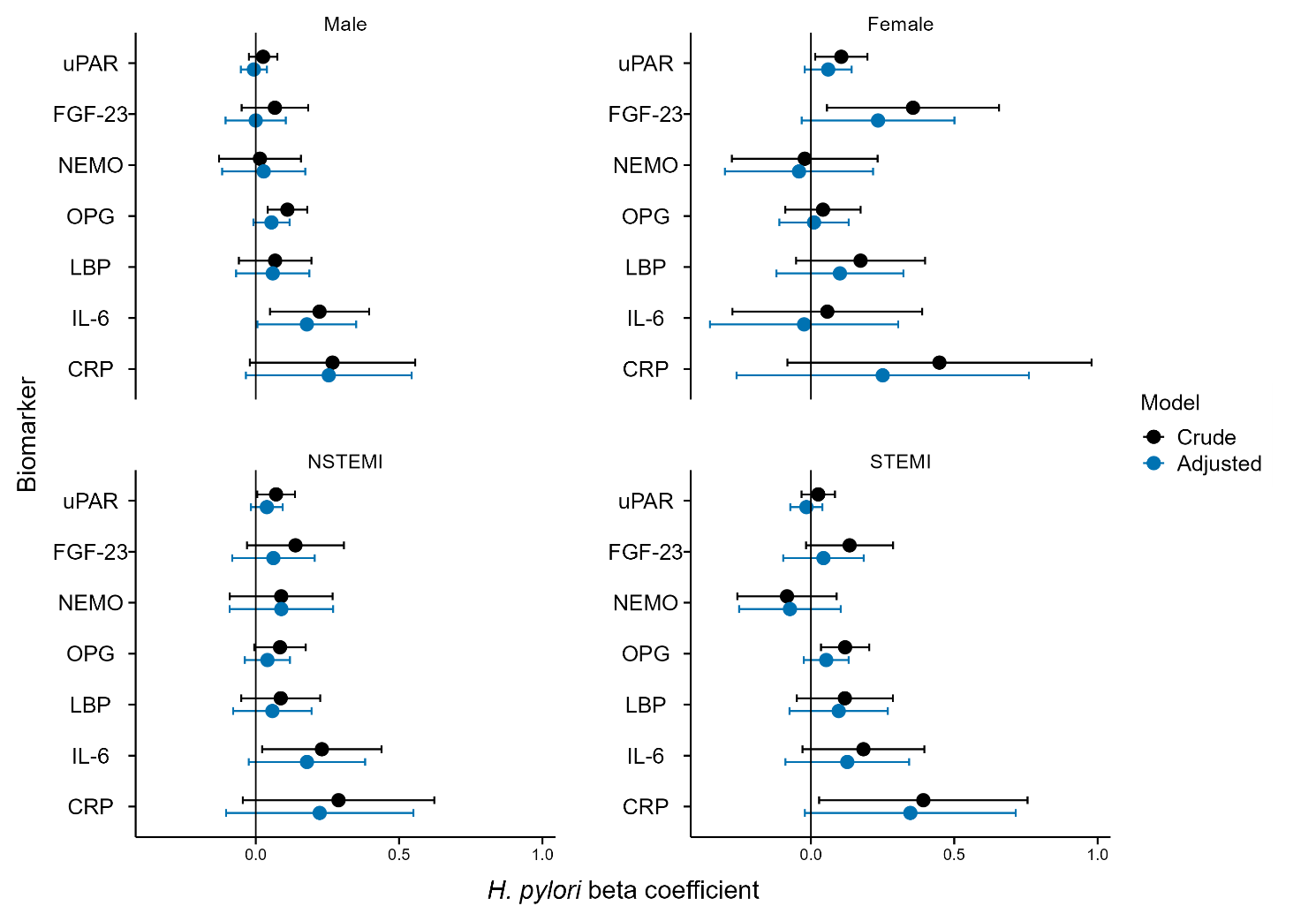


Supplemental Figure 5. Subgroup analysis of preselected biomarkers by sex and myocardial infarction (MI) type in all *H. pylori* positive patients. Beta-coefficients for *H. pylori* positive patients used in linear regression for each pre-specified biomarker with 95% confidence intervals. A crude model and an adjusted model are presented, which include sex, age, current smoking, hypertension, diabetes mellitus, eGFR, BMI, and hyperlipidemia as covariates. uPAR: urokinase-type plasminogen activator receptor, NEMO: nuclear factor-κB essential modulator, OPG: osteoprotegerin, FGF-23: Fibroblast growth factor 23, LPB: lipopolysaccharide binding protein, IL-6: interleukin-6, CRP: C-reactive protein, STEMI: ST-elevation MI, NSTEMI: non-ST-elevation MI.


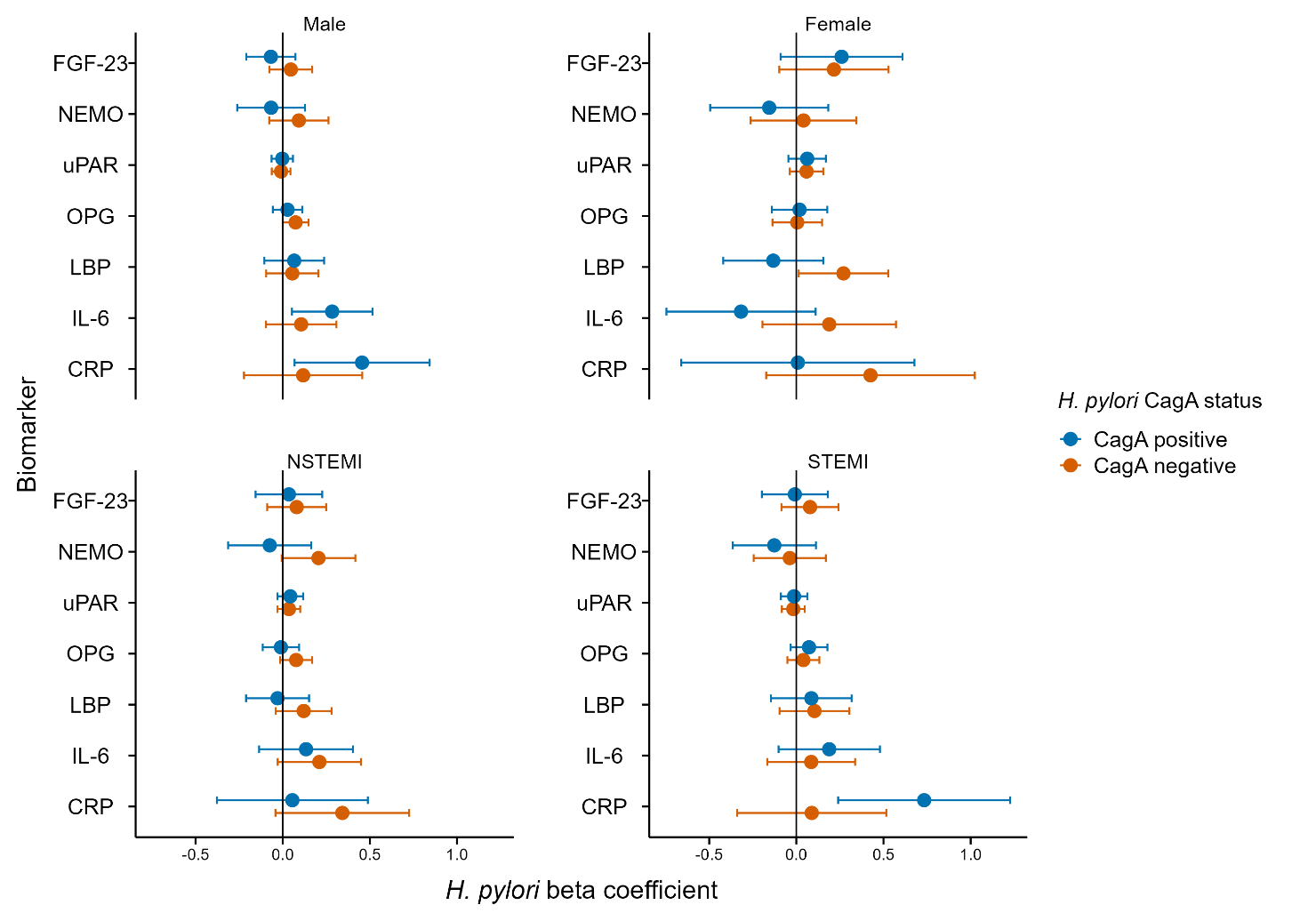


Supplemental Figure 6. Subgroup analysis of preselected biomarkers by sex and myocardial infarction (MI) type in *H. pylori* positive patients stratified by CagA status. Beta-coefficients for
*H. pylori* CagA positive and *H. pylori* positive CagA negative patients were used in linear regression for each pre-specified biomarker with 95% confidence intervals. Models were adjusted for sex, age, smoking, hypertension, diabetes mellitus, eGFR, BMI and hyperlipidemia. uPAR: urokinase-type plasminogen activator receptor, NEMO: nuclear factor-κB essential modulator. OPG: osteoprotegerin, FGF-23: Fibroblast growth factor 23, LPB: lipopolysaccharide binding protein, IL-6: interleukin-6, CRP: C-reactive protein, STEMI: ST-elevation MI, NSTEMI: non-ST-elevation MI, CagA: Cytotoxin-associated gene A.


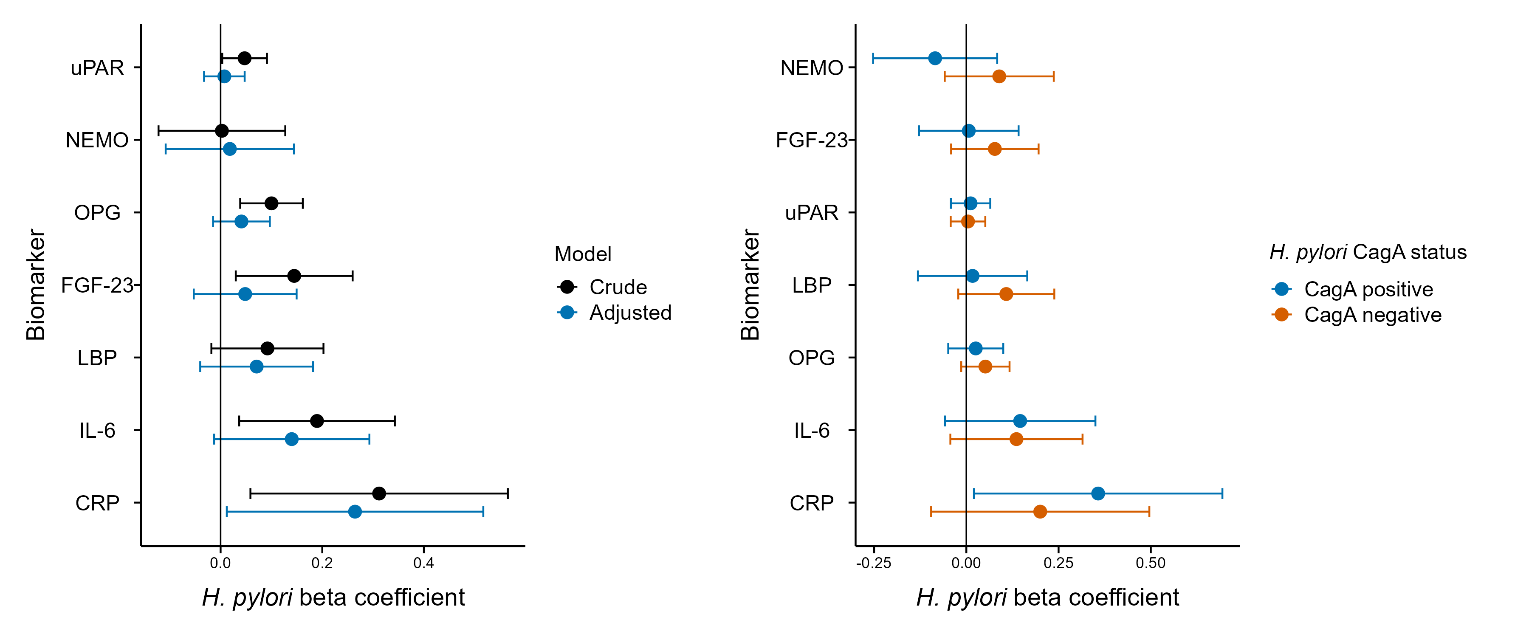


Supplemental Figure 7. Post-hoc sensitivity analysis of prespecified biomarkers in patients with MI in *H. pylori* groups and Cytotoxin-associated gene A (CagA) groups, additionally adjusted for angiotensin-converting enzyme inhibitors or angiotensin receptor blockers on admission.


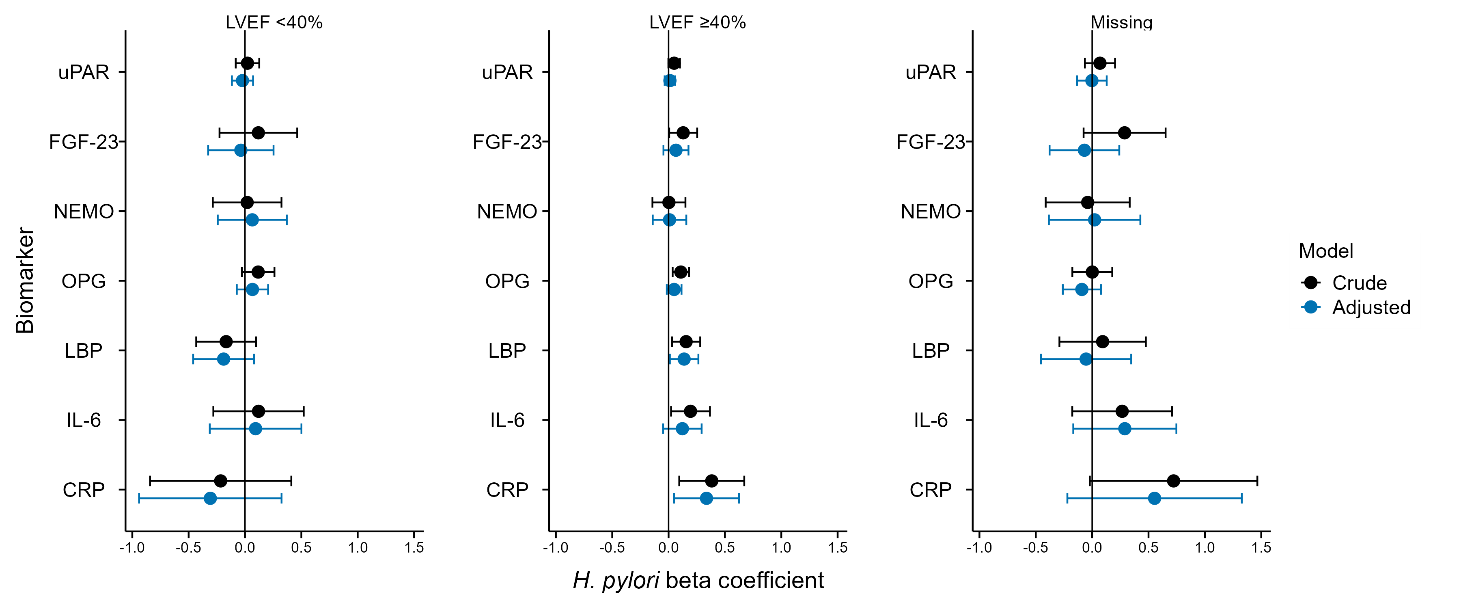


Supplemental Figure 8. Post-hoc subgroup analysis of preselected biomarkers by left ventricular ejection fraction (LVEF) groups in *H. pylori* positive vs negative patients with MI.


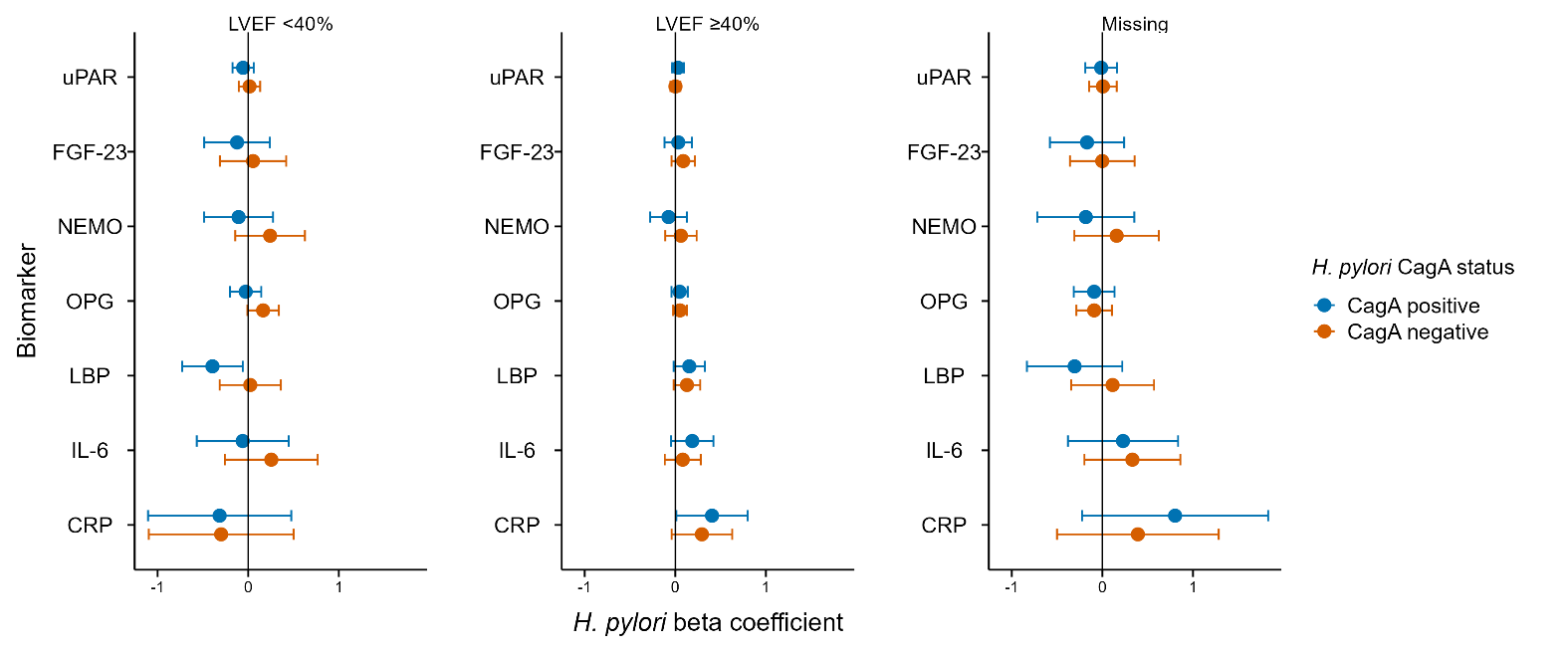


Supplemental Figure 9. Post-hoc subgroup analysis of preselected biomarkers by left ventricular ejection fraction (LVEF) groups in patients with MI with *H. pylori* Cytotoxin-associated gene A (CagA) groups compared to *H. pylori* negative.


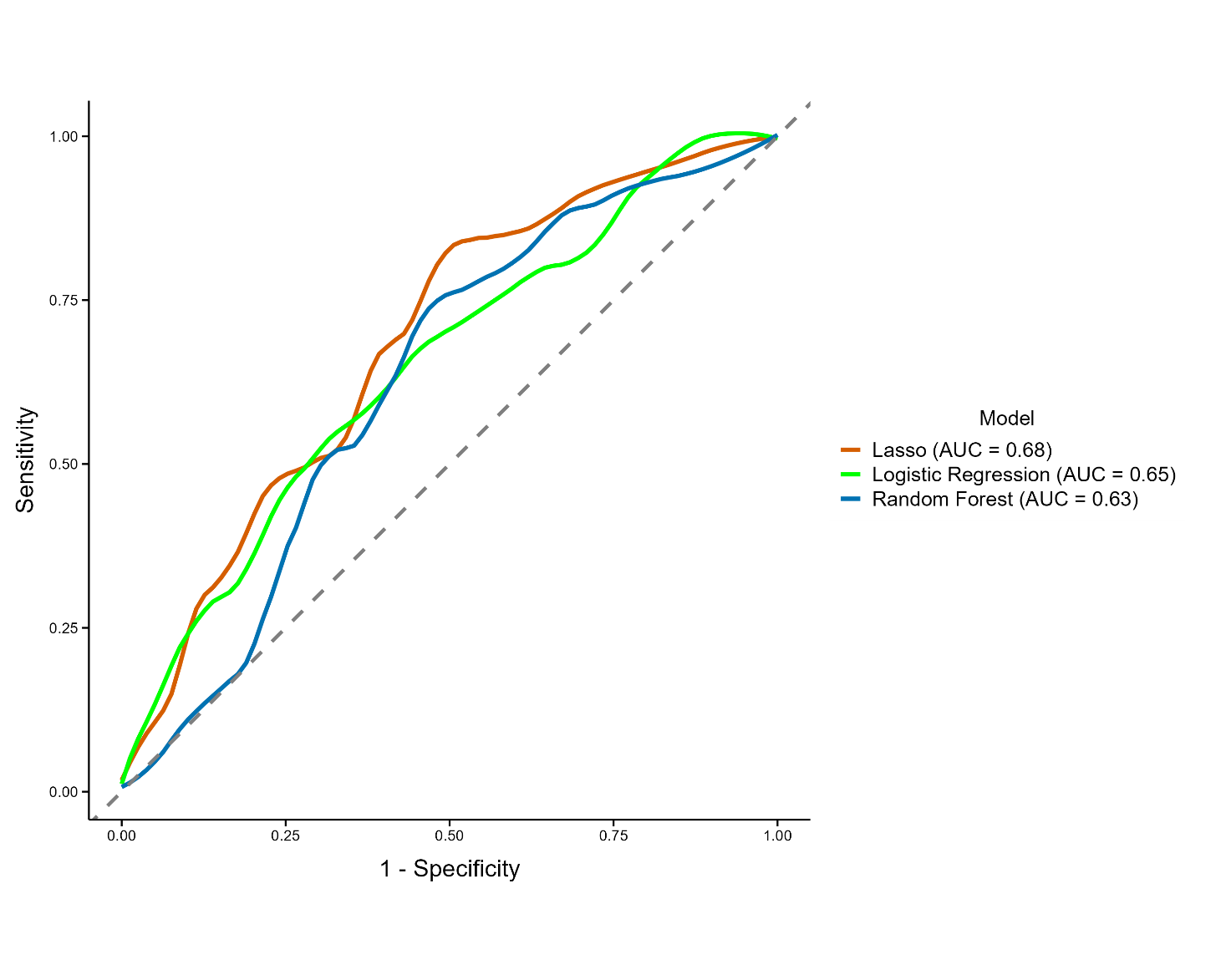


Supplemental Figure 10. Prediction of *H. pylori* status in patients with myocardial infarction using biomarkers and clinical data. Models were trained to predict *H. pylori* seropositivity using 175 biomarkers and clinical data on 80% of the dataset using five-fold cross-validation. Final models were evaluated on the remaining 20% testing data. Results are presented as receiver operating characteristic (ROC) curves, and area under the curve (AUC) values are shown for random forest and lasso regression, which were compared to a regular logistic regression model.


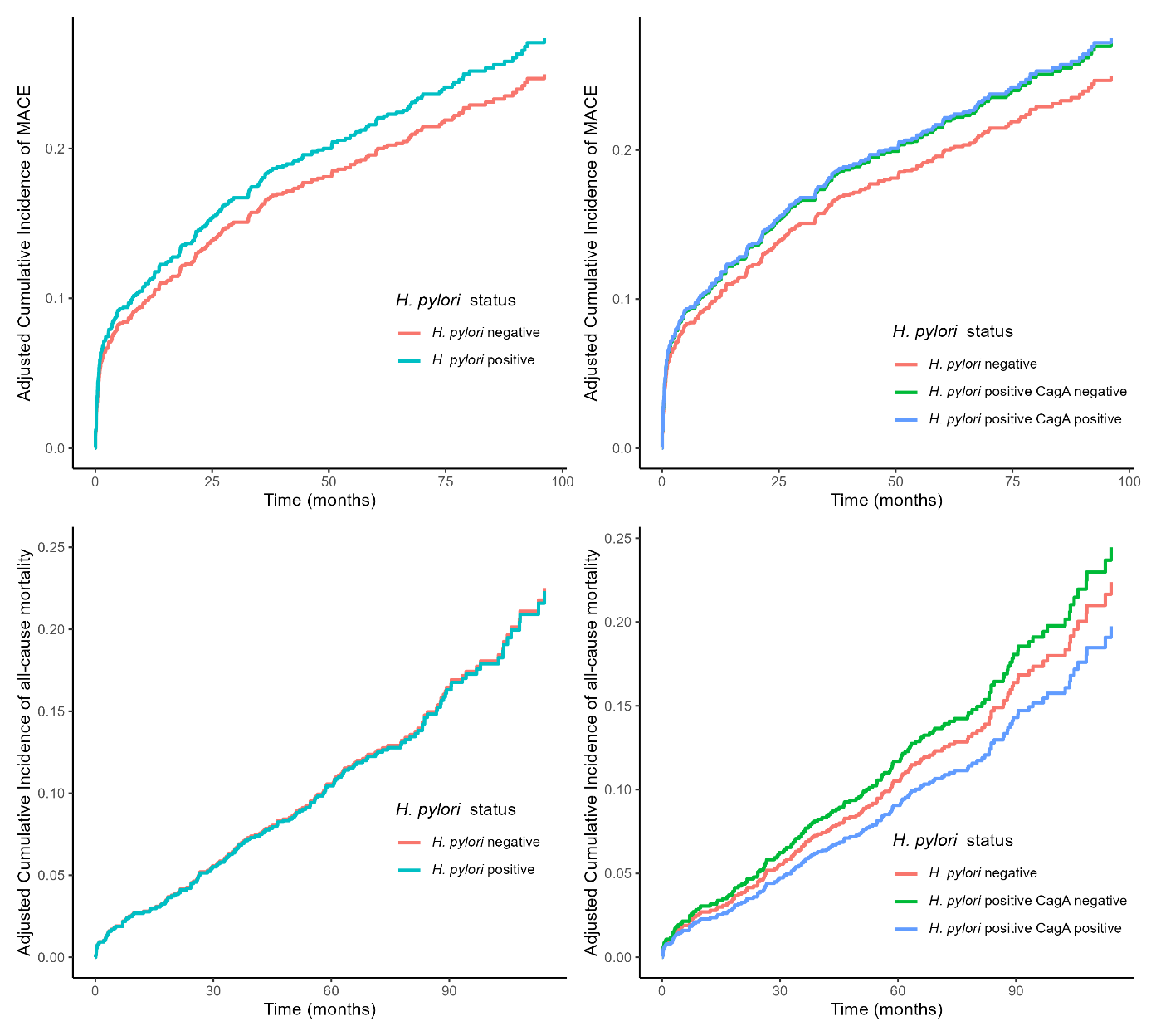


Supplemental Figure 11. Adjusted cumulative incidence plot of the association between *H. pylori* and CagA serology with Major adverse cardiovascular events (MACE) and all-cause mortality after MI. Cox-regression models were adjusted for sex, age, smoking, hypertension, diabetes mellitus, eGFR, BMI, hyperlipidemia, ST-elevation MI or non-ST-elevation MI, year of MI, and a previous diagnosis of chronic obstructive pulmonary disease, MI, heart failure, stroke, and gastrointestinal bleeding. CagA: Cytotoxin-associated gene A.


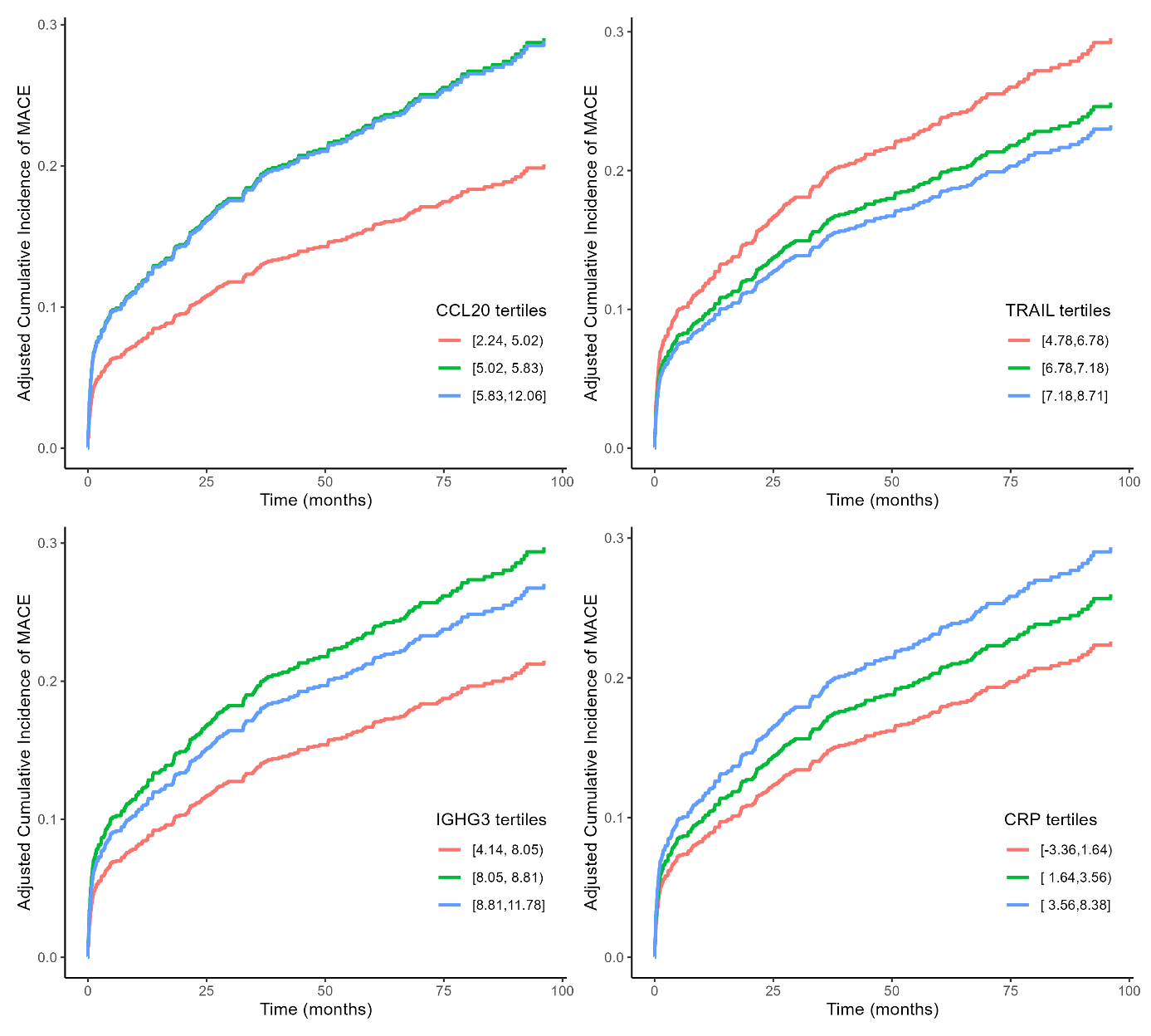


Supplemental Figure 12. Adjusted cumulative incidence plot of the association between biomarkers that were different in *H. pylori* positive patients and major adverse cardiovascular events (MACE). Cox-regression models were adjusted for sex, age, smoking, hypertension, diabetes mellitus, eGFR, BMI, hyperlipidemia, ST-elevation MI or non-ST-elevation MI, year of MI, left ventricular ejection fraction, and a previous diagnosis of chronic obstructive pulmonary disease, MI, heart failure, and stroke. TRAIL: TNF-related apoptosis-inducing ligand, CCL20: C-C motif chemokine ligand 20, IGHG3: immunoglobulin heavy constant gamma 3, CRP: C-reactive protein


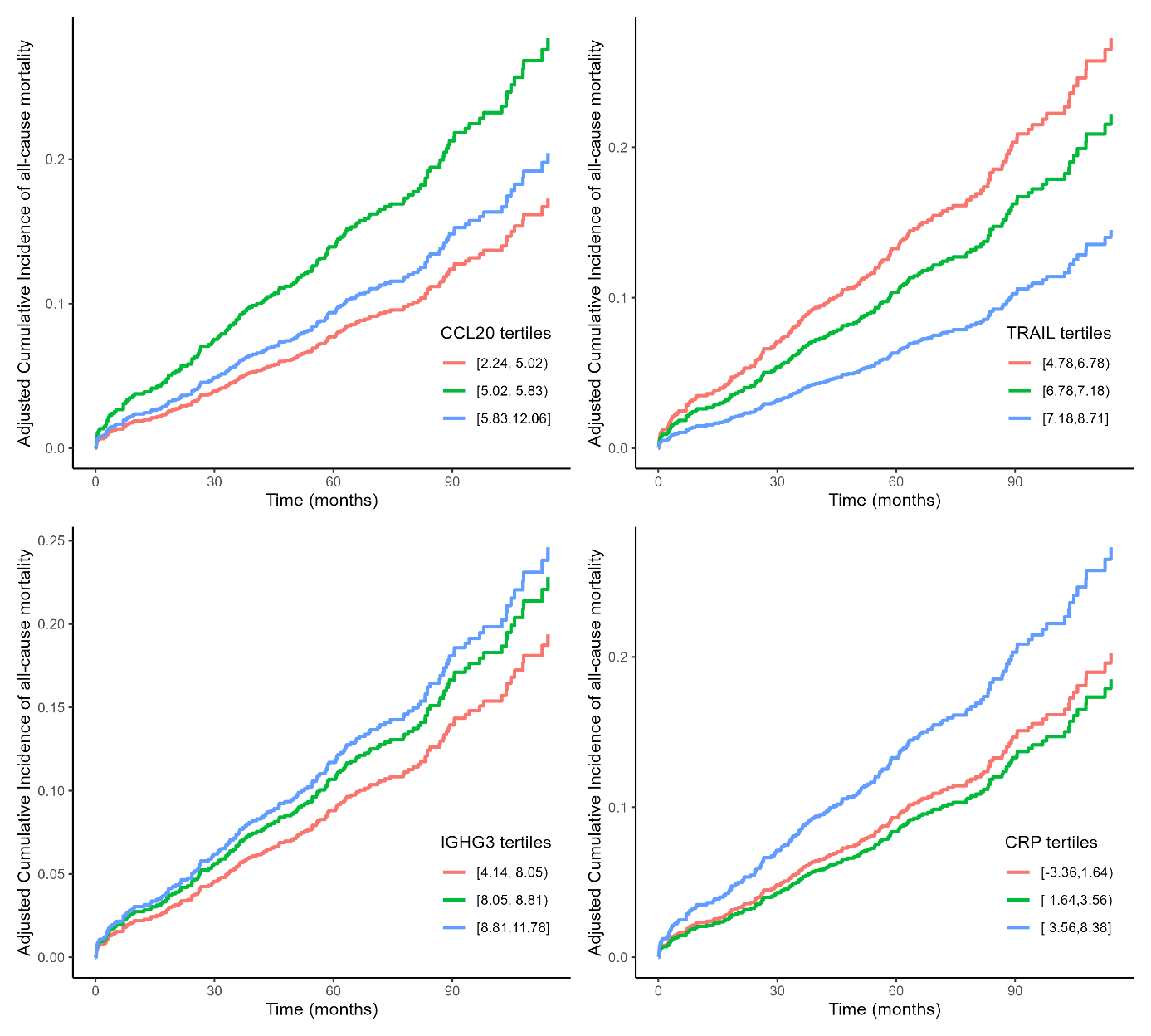


Supplemental Figure 13. Adjusted cumulative incidence plot of the association between biomarkers that were different in *H. pylori* positive patients and all-cause mortality. Cox-regression models were adjusted for sex, age, smoking, hypertension, diabetes mellitus, eGFR, BMI, hyperlipidemia, ST-elevation MI or non-ST-elevation MI, year of MI, left ventricular ejection fraction, and a previous diagnosis of chronic obstructive pulmonary disease, MI, heart failure, and stroke. TRAIL: TNF-related apoptosis-inducing ligand, CCL20: C-C motif chemokine ligand 20, IGHG3: immunoglobulin heavy constant gamma 3, CRP: C-reactive protein
